# Classifying Breast Cancer Metastasis Based on Imaging of Tumor Primary and Tumor Biology

**DOI:** 10.3390/diagnostics13030437

**Published:** 2023-01-25

**Authors:** Barbara Awad, Agni Chandora, Ben Bassett, Brittany Hermecz, Stefanie Woodard

**Affiliations:** 1 Debusk College of Osteopathic Medicine, Lincoln Memorial University, 6965 Cumberland Gap Pkwy, Harrogate, TN 37752, USA; 2 Department of Radiology, The University of Alabama at Birmingham, 619 19th Street South, Birmingham, AL 35249, USA; 3 Vulcan Imaging Associates, 2204 Lakeshore Dr. #140, Birmingham, AL 35209, USA

**Keywords:** breast cancer, molecular biology, metastatic breast cancer, luminal, TNBC, HER2+, breast MRI, breast ultrasound, mammography

## Abstract

The molecular classification of breast cancer has allowed for a better understanding of both prognosis and treatment of breast cancer. Imaging of the different molecular subtypes has revealed that biologically different tumors often exhibit typical features in mammography, ultrasound, and MRI. Here, we introduce the molecular classification of breast cancer and review the typical imaging features of each subtype, examining the predictive value of imaging with respect to distant metastases.

## 1. Introduction 

The histological classification of breast cancer along with the Nottingham grading system (NGS) have provided a framework for molecular classification of breast cancer and have long been used in conjunction with image interpretation. According to histologic classification, breast cancer can be divided into several subtypes. Examples of histological categorization include the following: invasive ductal carcinoma of no special type (IDC-NST), invasive lobular carcinoma (ILC), medullary carcinoma, metaplastic carcinoma, apocrine carcinoma, mucinous carcinoma, cribriform carcinoma, tubular carcinoma, and neuroendocrine carcinoma [1].

As breast cancer becomes better understood through molecular classification, the understanding of imaging has evolved. Molecular classification divides breast cancer into four major subtypes. These subtypes include luminal A, luminal B, basal-like or triple- negative breast cancer (TNBC), and human epidermal growth factor receptor 2 (HER2+) [2]. Each subtype has different characteristics. For example, one of the determinants of subtype is Ki-67, which is a proliferative marker for breast cancer. Ki-67 has been found to significantly correlate with the incidence of metastasis and recurrence. The cut-off values of Ki-67 may vary. In general, a higher Ki-67 value correlates with greater tumoral metastasis and poor prognosis. A Ki-67 of 15% is often considered a low value [3]. 

Luminal-type breast cancer is the most common type and is divided into two subtypes, luminal A and luminal B. This subtype is defined as having a low Ki-67 in comparison to hormonal receptor (HR)-negative breast cancer subtypes, which are HER2+ and basal-like, that have higher Ki-67 indexes and are, therefore, higher-grade tumors [4]. These subtypes and their characteristics are presented in Table 1 from do Nascimento and Otani [1]. The molecular subtypes not only correlate with the proliferative potential of each tumor, but they are linked with different prognostic outcomes.

Through genomic expression profiling studies and advances in imaging, the breast cancer classification system is now based on tumor biology instead of tumor morphology. In addition, the metastasis pattern now correlates to a greater degree with the molecular classification of the subtypes of breast cancer. It has been proven that several imaging features can aid in prediction and diagnosis of these subtypes, ultimately leading to better treatment outcomes. Although histopathological characterization from a breast biopsy is the gold standard for breast cancer classification, imaging remains critical due to its ability to diagnose in a rapid and noninvasive manner [5]. Primary imaging modalities are typically mammography and ultrasound. Previous studies indicate that mammographic appearance could be correlated with molecular subtype and biologic behavior of breast tumors. Various mammographic characteristics have been shown to correlate with less aggressive (luminal A and B) and more aggressive (basal-like, HER2+) subtypes [6].

Ultrasound has shown promise in disease assessment, especially when combined with mammography [7]. However, more advanced imaging modalities, such as breast MRI, provide true physiologic assessment. Even non-contrast MRI has shown promise. For example, T2-weighted sequences increase the specificity of MRI in the assessment of tumoral necrosis, which is commonly seen in the aggressive phenotype of triple-negative breast cancer (TNBC). In addition, the discovery of peritumoral edema is a characteristic of poor prognosis (more common with basal-like), and is observed effectively with T2-weighted imaging [8,9,10]. Lastly, full-body imaging provides both vascular and metabolic information that aids in differentiation of subtype [11]. This review will provide an imaging overview of the different molecular subtypes of breast cancer and their common metastatic locations and appearances.

## 2. Materials and Methods

A literature review was conducted using terminology regarding molecular subtypes and imaging modalities as key words. Initially, the search was limited to studies completed within the last five years, and then broadened to include texts of earlier years that were reviewed in the articles’ references. Articles that focused on treatment modalities rather than imaging and subtype correlation were excluded from this review. Additionally, articles that concentrated on noninvasive disease (ductal carcinoma in situ, DCIS) were also excluded.

## 3. Results

### 3.1. Luminal A

The luminal A breast cancer subtype comprises 50% of invasive breast cancers and has an overall good prognosis. This subtype is estrogen receptor-positive (ER+), progesterone receptor-positive (PR+) and HER2/neu-negative. Luminal A exhibits a high expression of hormone receptors and hormone-related genes [2]. On average, luminal A tumors are typically seen in older women compared to TNBC.

#### 3.1.1. Imaging Findings

##### Mammography and Ultrasound

Spiculation seen on mammography is a characteristic finding of invasive breast cancer and is a strong predictor of malignancy. Spiculated masses are defined as masses with margins that have lines radiating from them, versus non-spiculated masses, which have circumscribed, microlobulated, or indistinct margins [12]. In a study by S. Liu et al., spiculated margins were significantly more common in patients with luminal A breast cancer; however, Shaikh et al. found that HER2+ cancers and luminal B also showed spiculation [5]. The exact mechanism of spiculation remains unknown, but there is a strong correlation of spiculation in tumors that have a low Ki-67 index [13], in tumors that have significantly higher ER+ and PR+ rates [14], and in HER2- tumors [15]. Interestingly, Sartor et al. studied the genetic associations of tumors with a predominant spiculated appearance vs. tumors with a predominant mass appearance. They concluded that genes expressing spiculations were enriched with extracellular matrix genes, which matches with the known desmoplastic appearance of spiculations. On the contrary, genes expressing masses were more enriched with proliferation. By determining the differences in genes between the spiculated and mass-tumor groups, they described a spiculated metagene that correlated with improved survival in a specific subset of grade 2 tumors [16].

Ultrasound characteristics such as margins, size, vascularity, posterior enhancement, and shadowing have been investigated for subtype association. Non-circumscribed margins with posterior shadowing were associated with the less aggressive subtypes (luminal A and B) [6]. Ian et al. demonstrated that luminal subtypes often present with spiculated margins and posterior acoustic shadowing in sonography [17].

##### MRI

According to a multivariate analysis in 2022, luminal A breast cancer subtypes are often smaller and do not show rim enhancement in MRI [8]. With regard to the features of a mass in MRI, irregular margins and a non-round shape in MRI were significantly associated with luminal A [18]. Luminal A was least likely of all the subtypes to have multifocality/multicentricty or lymph node involvement, found in only 27.3% and 17.3% of cases, respectively, according to Grimm et al. [19].

Use of machine learning (ML) in MRI has also supported subtype differentiation, based on extracted features in luminal A type breast cancer, with an accuracy of 89.2% when combined with ML pathologic features and 69.9% based on imaging ML alone [20]. Computer vision algorithms used to extract numerous imaging features from breast MRI found that luminal A could be accurately predicted based on certain features, including a two-timepoint ratio of tumor enhancement to fibroglandular background and the specific sequence of peak enhancement [21]. Kato et al. also found that MRI can be useful in clinically node-negative luminal A cases. They investigated the utility of assessing the ADC of luminal A cancers, determining that a minimum ADC value of the breast lesion had a high sensitivity for axillary nodal metastases. In addition, they found a high negative predictive value (NPV) and suggested potential future omission of sentinel node biopsy procedures based on a higher minimum ADC breast lesion value [22].

##### Imaging and Metastases

Metastatic disease from luminal A breast cancer most commonly occurs in bone and can be seen 5–10 years after the original diagnosis, with some studies documenting metastases up to 20 years later [23,24]. Numerous guidelines exist to help clinicians choose the appropriate imaging for metastases; however, there is no single consensus [11]. Bone scintigraphy (BS) is the imaging modality of choice in patients with concern for osseous disease. Single-photon emission computerized tomography (SPECT) can be added to BS for a higher specificity [25]. CT of the chest, abdomen, and pelvis can also be performed if there is concern for metastasis due to abdominal–pelvic pain or abnormal tumor marker values (elevated CA 15.3) [24,26].

Patients with advanced disease, such as those with T4 tumors and a large axillary node burden, may also be considered for metastatic surveillance [11]. Advanced imaging for detecting ER-positive tumors includes Fluorine-18-radiolabeled fluoroestradiol (^18^F-FES), which can also assess the probability of therapeutic success. Radiolabeled pharmaceuticals with positron emission tomography (PET) and labeling of estradiol/analogues with SPECT can also aid in assessment of ER positive tumors. PR imaging has also been studied with some success. Cell proliferation imaging with fluorine-18 fluorothymidine PET can aid in differentiating luminal A from luminal B tumors, since this modality correlates with Ki-67 [27]. While ^18^F-fluorodeoxyglucose (FDG) PET/CT is not a standard imaging tool in luminal A cancers, it has shown promise in differentiation of molecular subtypes of cancer based on varied standard uptake values (SUVs). The largest isolated semi-quantitative SUV difference was found between luminal A and basal subtypes, as luminal A generally demonstrated the lowest SUV values [28]. With regard to luminal subtypes, SUVmax of metastatic breast cancer on PET/CT may be a better predictor of progression-free survival (PFS) and overall survival (OS) than the molecular subtype at the time of patient’s diagnosis [29].

#### 3.1.2. Treatment

The mainstay of all breast cancer treatment includes a combination of surgery, radiation, and/or systemic therapy. The type of systemic therapy utilized offers the greatest variability in treatment between the different subtypes of breast cancer, as efficacy is more type-specific compared to surgery and radiation. Luminal A expresses hormone receptors leading to good response to endocrine therapy; however, this subtype’s response to chemotherapy is variable [2,30]. Patients often receive endocrine therapy following surgical excision for five to ten years. Primary endocrine therapy is an option in cases where there are contraindications to surgery or downstaging is necessary in order to undergo breast conservation surgery instead of mastectomy [31].

### 3.2. Luminal B

Luminal B breast cancer subtypes make up 20% of invasive breast cancers expressing moderate to low amounts of hormone receptors in comparison to luminal A. Luminal B is ER+ and PR−/+. In comparison to luminal A, luminal B has variable expression of HER2/neu expression and has a higher proliferation index with moderate differentiation (grade II) [2].

#### 3.2.1. Imaging Findings

##### Mammography and Ultrasound

Ultrasound and mammography imaging for luminal B is very similar to luminal A, although certain features may help to differentiate the two. Mammographic features have not often been studied as independent imaging predictors of subtype, except for microcalcifications, which typically do not differentiate luminal B. However, some studies have found microcalcifications to be predictors of HER2+ tumors in general, whether luminal B or HER2+ [5,6]. A meta-analysis of imaging features in association with molecular subtypes found that even the morphology of microcalcifications can be indicative. Fine linear/fine linear branching microcalcifications, which are the morphologies with the highest positive predictive value for malignancy, strongly correlated with HER2+ disease (luminal B, HER2+). Breast density also had a higher association with HER2 positivity [32]. On ultrasound, increased internal vascularity has been associated with luminal B when other features (not-circumscribed margins, posterior acoustic shadowing) are present [6]. Circumscribed margins seen in this subtype are associated with a decreased chance of overexpression of HER2 [32].

##### MRI

On MRI, a round morphology is often associated with the luminal B subtype. Rim enhancement is not typically seen [8]. This lack of rim enhancement is similar to luminal A. In a study by Grimm et al., luminal B masses are most commonly described by both irregular margins and irregular morphology. When associated with non-mass enhancement, the internal enhancement pattern was never homogeneous [33]. Just as with luminal A, computer vision algorithms extracting features from MRI found that the two-timepoint ratio of tumor enhancement to fibroglandular background and the specific sequence of peak enhancement correlated well with the luminal B subtype [21]. 

##### Imaging and Metastases

Luminal B breast cancer has a similar metastatic imaging profile compared to luminal A. As discussed with regard to luminal A subtype, cell proliferation imaging with fluorine-18 fluorothymidine PET can aid in diagnosis of luminal B due to its strong association with Ki-67 (higher uptake would be more indicative of a luminal B tumor rather than luminal A) [27]. PET/CT has also been shown to both aid in detection of luminal B type metastases and more accurately predict OS than circulating cancer antigen (CA) 15.3 levels, according to Urso et al. [34]. Just as with luminal A, ^18^F-FDG PET/CT is not a standard imaging tool for luminal B cancers; however, increased SUV uptake is found in more aggressive luminal B tumors [28]. PET/CT imaging of metastatic disease in luminal B patients may more accurately predict PFS and OS, similar to luminal A [29].

#### 3.2.2. Treatment

Similar to luminal A, luminal B does respond to endocrine therapy, though to a lesser degree [2]. Luminal B subtype is a heterogenous group and, thus, has a variable response rate to chemotherapy based on differing levels of gene expression [35]. This subtype, however, does respond better to chemotherapy in comparison to luminal A [36]. In certain cases, neoadjuvant chemotherapy may be utilized for downstaging. Because of the comparatively increased response to chemotherapy, patients who receive neoadjuvant chemotherapy achieve pathologic complete response at a higher rate than patients with luminal A type, albeit at a lower rate compared to HER2+ and TNB [30]. 

### 3.3. Basal-like

Basal-like breast cancers are typically classified as TNBC because these tumors lack expression of ER, PR, and HER2/neu. Although the terms triple-negative and basal-like have been interchangeable, the two can be distinguished in that not all triple-negative tumors have gene expression that is basal-like. In addition, not all basal-like breast cancers are triple-negative [37]. Although it is important to note these differences, for the purposes of this manuscript the two terms will not be distinguished. Basal-like tumors are typically high-grade and highly proliferative, and account for 15% of invasive breast cancer [18]. 

#### 3.3.1. Imaging Findings

##### Mammography and Ultrasound

On mammography, basal-like breast cancers are often larger than other subtypes and have circumscribed margins [5]. On ultrasound, mass morphology is often oval or round with circumscribed margins, internal echogenicity is hypoechoic, and there is typically posterior acoustic enhancement. Caution must be exercised with such features since there is overlap between common, benign lesions such as complicated cysts, fibroadenomas, or hematomas. Using a size cut-off for solid masses with such benign-appearing features can help determine whether to recommend a biopsy [5].

##### MRI

Youk et al. described the features of TNBC on MRI, finding that round or oval morphologies were most common. TNBC also tended to be larger, more defined, and more necrotic than other subtypes of breast cancers [38]. Rim enhancement, corresponding to central necrosis, was the most commonly reported descriptor of TNBC in a systematic review from 2021. In addition, kinetics often demonstrate type 3 features with rapid uptake and washout of contrast. As opposed to all other subtypes, TNBC tumors are often described as circumscribed, a term typically indicative of a benign lesion [39]. This characteristic has been a defining feature of the TNBC subtype. Galati et al. also reviewed breast MRI features of different molecular subtypes, finding a strong association between triple-negative tumors and the following characteristics: round morphology, intralesional necrosis, rim enhancement, and peritumoral edema [8]. 

##### Imaging and Metastases

Imaging for metastatic disease is not part of a standard work-up for basal-like breast cancer; however, this subtype is the most likely to metastasize. In addition, metastases may occur early (less than a year). After metastases occur, the median survival is about 0.5 years, but if a patient is free of metastases by 7 years after diagnosis, the disease-free survival (DFS) significantly increases. Thus, imaging for early distant metastasis in basal-like cancers is more important compared to luminal types, which tend to metastasize later. Since these tumor types are less likely to have locoregional disease recurrence versus metastases, more frequent imaging of the breast tissue itself may not be as important. Metastases more commonly involve the CNS and lung, occurring less commonly in the bone and liver [24]. Therefore, when imaging for metastatic disease in basal-like cancers, earlier full-body imaging with attention to lung and brain may be most fruitful. ^18^F-FDG uptake shows significantly higher uptake in TNBCs compared to other subtypes, and this finding holds true both with lymph node disease and with organ metastases [27,40]. 

#### 3.3.2. Treatment 

Since TNBC and basal-like subtypes lack ER, PR, and HER2/neu expression, they do not respond to endocrine therapy or trastuzumab [2]. However, these subtypes are susceptible to chemotherapy, with front-line therapy including taxane and anthracycline [41]. While neoadjuvant chemotherapy was previously reserved for non-operable tumors, it has now become standard care for treatment in TNBC [31]. Response to preoperative therapy is used to guide surgical planning and postoperative treatment [42], with some patients achieving favorable results allowing them to de-escalate from mastectomy to breast-conserving surgery [43]. 

### 3.4. Human Epidermal Growth Factor Receptor Type 2 Positive (HER2+)

Human epidermal growth factor receptor type 2 (HER2) proteins are receptors on breast cells that normally help control how a healthy breast cell grows, divides, and repairs [44]. HER2+ breast cancers constitute 15% of invasive breast cancer. This subtype is highly proliferative and classified as ER negative, PR negative, and HER2/neu positive. HER2+ tumors also have frequent nodal involvement and a high histological grade. According to the American Cancer Society, all invasive breast cancers should be tested for HER2, either by biopsy or when the tumor is surgically removed, due to its highly proliferative nature. HER2+ overexpression is associated with increased vascularity in tumors due to the increase in vascular endothelial growth factor (VEGF) production [45]. 

#### 3.4.1. Imaging Findings

##### Mammography and Ultrasound 

Microcalcifications are widely thought to be associated with HER2+ breast cancers. Furthermore, the mere presence of microcalcifications, as well as increased breast density, both correlate with an increased chance of overexpression of HER2 [32]. Suspicious microcalcifications and microlobulated margins, as well as hypoechoic masses with increased internal vascularity, are predictive of HER2-enriched cancers. Internal vascularity is correlated with highly invasive cancer tumors [17]. HER2+ breast cancer is very strongly related to the presence of microcalcifications with or without a visualized mass [5]. Jiang et al. found that tumors with calcifications had a higher correlation with axillary nodal metastasis, and in their study (investigating IDC without specific delineation of HER2+ cancers), an association with HER2+ may be extrapolated, given the strong association of calcifications with this subtype [14]. 

##### MRI

Breast MRI is not specifically recommended for HER2+ disease assessment, but there are certain tumor features indicative of a HER2+ subtype in MRI. Features shown to be associated with HER2+ tumors include washout kinetics and rapid uptake of contrast. Other features that have been associated with HER2+ include irregular margins and multifocality, which may be a reason for considering routine use of MRI [32]. Round margins, hyperintense T2 signal, and rim enhancement were associated with HER2+, according to Ozturk et al. [46]. A systematic review by Ab Mumin et al. noted that irregular and round morphology were more common with HER2+ tumors. Spiculated and non-circumscribed were the margin descriptions most often associated with HER2+ tumors. In this review, hypointense T2 signal was associated with HER2+. The internal enhancement pattern description was most often reported as heterogeneous, and the ADC values of HER2+ tumors were greater than all other subtypes [39]. 

##### Imaging and Metastases

Some of the greatest imaging advances impacting patient care are a result of advances in imaging of HER2+ disease. While HER2+ disease is known to be more aggressive and previously resulted in worse prognoses, targeted treatment has resulted in improved outcomes. Imaging has also progressed to match the biological advances in treatment. HER2-targeting agents such as alternative high affinity binders, monoclonal antibodies, immunoglobulin-based tracers, fragments of antibodies, and small non-immunoglobulin tracers have all been used for imaging HER2+ tumors [47,48]. In this regard, ^64^Cu-DOTA-trastuzumab PET, for instance, has been shown to effectively identify both HER2+ primary and metastatic lesions [49]. In addition, conventional PET/CT imaging of HER2+ tumors shows higher uptake than HER2- breast cancers and has been shown to be successful in identifying distant unsuspected metastases [32,50].

#### 3.4.2. Treatment

HER2+ subtype breast cancers are more likely to grow faster in comparison to other subtypes and have been associated with a poor prognosis; however, this subtype responds to treatment better, since treatments can specifically target the HER2 gene. Trastuzumab has been proven to aid in treatment of this subtype and has been used as a first-line chemotherapy agent in breast cancers that overexpress HER2+ [51]. A study conducted by Ferretti et al. further supported the conclusion that HER2+ overexpression can also aid in determining response to endocrine therapy or chemotherapy.

Supplemental material is provided to illustrate the imaging appearance of the primary and metastatic tumors discussed. Imaging is presented in a case-based format with Figures S1–S39 corresponding to the cases. Luminal A-type cases are shown by cases 1, 2, 3 and 4. The luminal B-type cases are illustrated by cases 5 and 6. Basal-like tumors can be seen in cases 7 and 8. Finally, the HER2+ tumors are presented in cases, 9, 10 and 11. Features of both the primary tumors and metastatic disease are discussed.

## 4. Conclusions

Although there is overlap in the imaging characteristics of the molecular subtypes of breast cancer and their respective metastases, some features can help differentiate between them. The heterogeneous nature of tumors and the sampling error of biopsies may limit pathologic evaluation; thus, imaging may provide needed information regarding a primary tumor. Occult disease, such as clinically negative nodes or unrecognized, asymptomatic metastatic disease, may also be detected during advanced imaging. Supplementing knowledge of tumor biology with appropriate, targeted imaging studies can provide the most thorough assessment and care for patients with breast cancer. A possible future direction for research may involve combining the imaging features delineated in this review with research in AI to provide direction for predicting tumor type, treatment, and prognosis.

## Figures and Tables

**Table 1:** Descriptive table of the molecular classification of breast cancer including the biomarkers, grade, prognoses, treatment, and frequency of cases. From do Nascimento RG, Otoni KM, “Histological and molecular classification of breast cancer: what do we know.” *Mastology*. 2020;30:e20200024, http://creativecommons.org/licences/by/4.0/, accessed on 6 November 2022 [1].

Molecular Subtypes	Luminal A	Luminal B		HER2+	TN
		(HER2−)	(HER2+)		
Biomarkers	ER+ PR+ HER2− Ki67low	ER+ PR− HER2− Ki67high	ER+ PR−/+ HER2+ Ki67low/high	ER− PR− HER2+ Ki67high	ER− PR− HER2− Ki67high
Frequency of Cases (%)	40–50	20–30		15–20	10–20
Histological Grade	Well Differentiated (Grade I)	Moderately Differentiated (Grade II)		Little Differentiated (Grade III)	Little Differentiated (Grade III)
Prognosis	Good	Intermediate		Poor	Poor
Response to Therapies	Endocrine	Endocrine Chemotherapy	Endocrine Chemotherapy Target Therapy	Target Therapy Chemotherapy	Chemotherapy PARP Inhibitors

## Supplementary Materials

The following are available online at https://www.mdpi.com/article/10.3390/diagnostics13030437/s1, Figures S1–S39 correspond to cases. Imaging is presented in a case format. Luminal A-type cases (cases 1, 2, 3, and 4), luminal B-type cases (cases 5, 6), basal-like (cases 7, 8) and HER2+ (cases, 9, 10, 11) demonstrate some of the features described of both primary and metastatic disease.
