# Supplementary material for: Classifying Breast Cancer Metastasis Based on Imaging of Tumor Primary and Tumor Biology
Source: Diagnostics (Basel). 2023 Jan 25;13(3):437. doi: 10.3390/diagnostics13030437 (PMC9914718; doi:10.3390/diagnostics13030437)
Supplement: Supplementary file 1 [file diagnostics-13-00437-s001.zip › diagnostics-2087816-supplementary.pdf]

Supplemental material: Imaging is presented in a case format. Luminal A type cases (cases 1, 2, 3, and 4), luminal B type cases (cases 5, 6), basal-like (cases 7, 8) and HER2+ (cases, 9, 10, 11) demonstrate some of the features described of both primary and metastatic disease. Figures 1-39 correspond to these cases.

CASE 1: 78-year-old female was diagnosed with left breast invasive ductal carcinoma (ER +, PR +, HER2-) and subsequently underwent lumpectomy and has been on aromatase inhibitors.

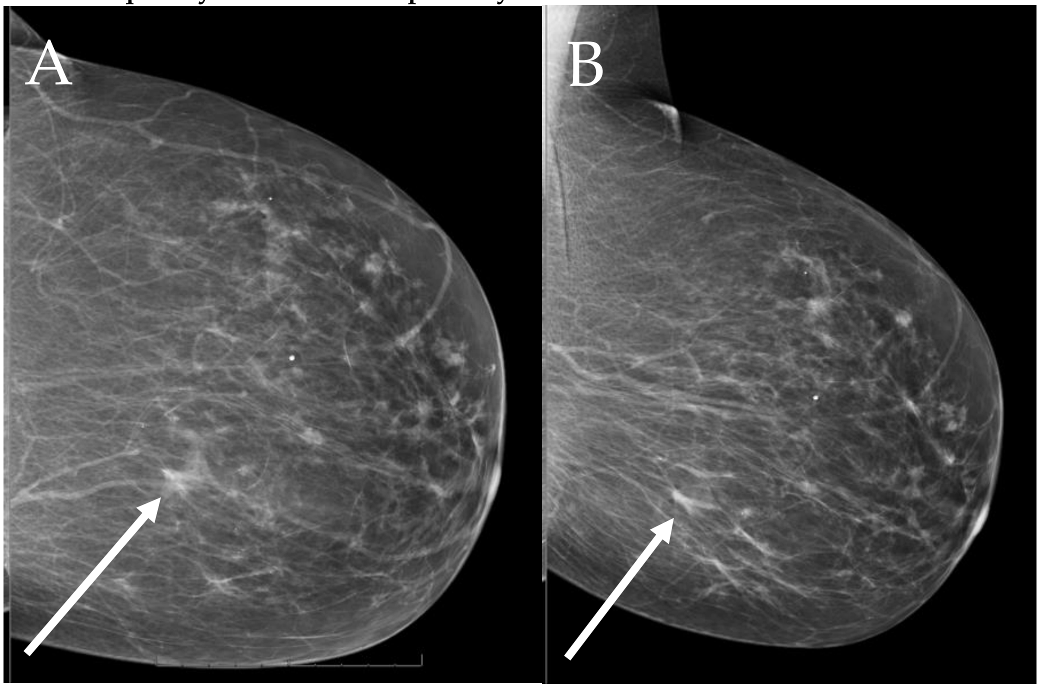

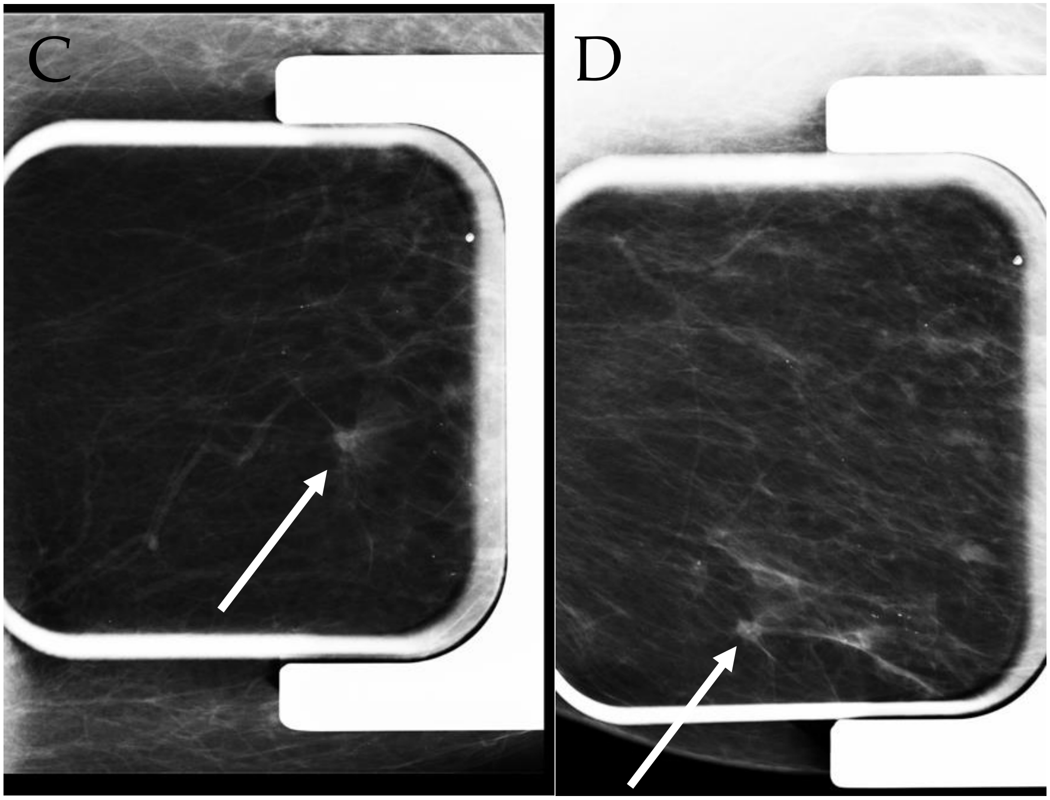

Figure S1. Left breast 2D CC (A) and MLO (B) views demonstrate a focal asymmetry (arrows) in the left breast 6:00 posterior depth. Dedicated spot views in CC (C) and MLO (D) were obtained, showing a spiculated mass.

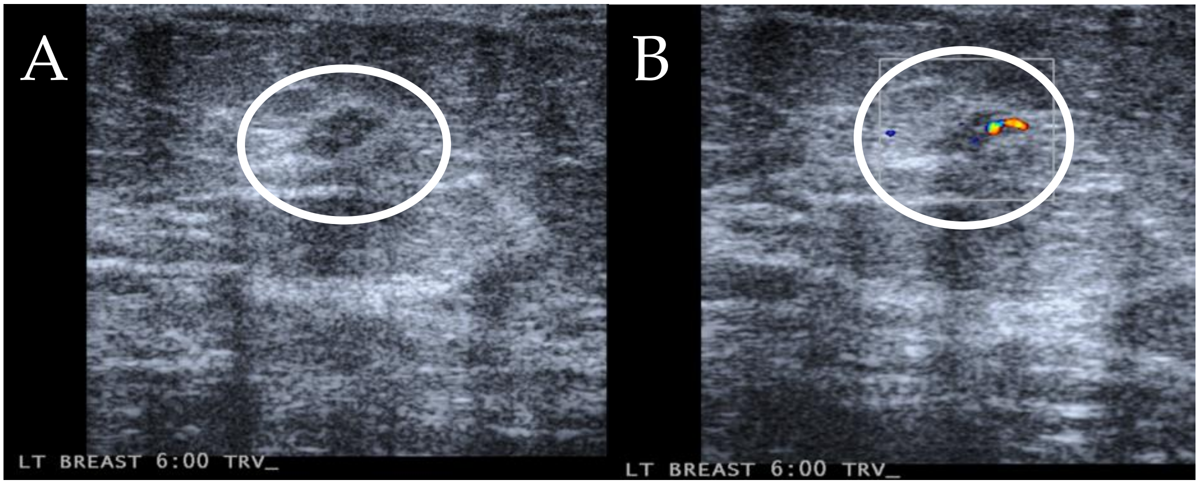

Figure S2. Left breast ultrasound, Grayscale (A) and Color Doppler (B) images in the transverse plane demonstrate a hypoechoic not circumscribed irregular mass with internal vascularity which measures 7 x 5 x 1 mm at 6:00 4cm from the nipple (circles).

CASE 2: 62-year-old female who presented with a palpable lump. She was diagnosed with invasive mucinous carcinoma of the right breast (ER+, PR+, HER2-).

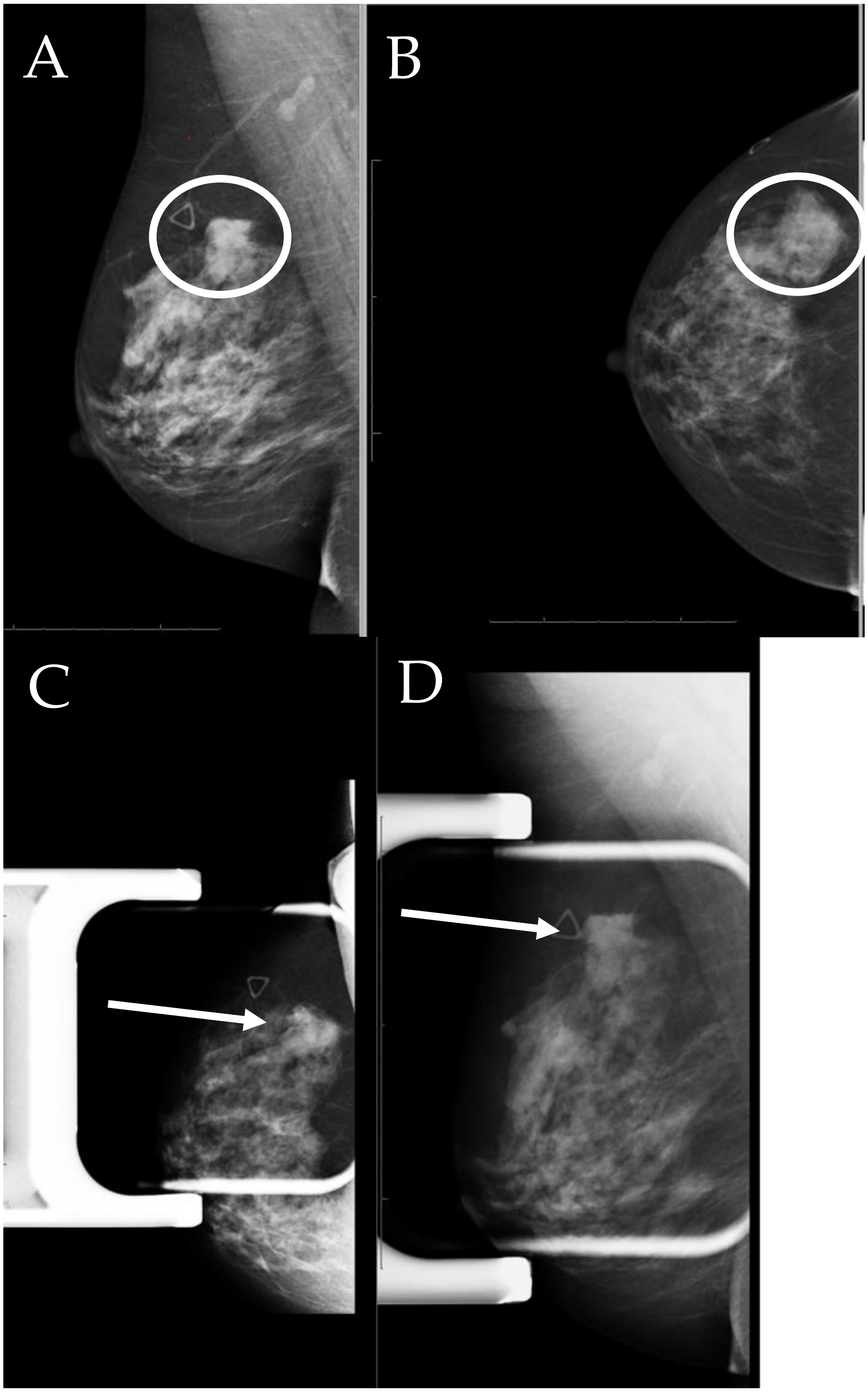

Figure S3. Right breast 2D MLO (A) and CC (B) full-field views as well as spot CC (C) and spot MLO (D) views at region of palpable abnormality were obtained which demonstrate a new irregular mass with spiculated margins seen in the right breast at 10 o'clock located 6 centimeters from the nipple (circles). This correlates to the palpable abnormality. There is also a new focal asymmetry seen in the right breast at 10 o'clock located 5 centimeters from the nipple (arrows). This focal asymmetry appears to be anterior to the larger mass on the mammographic images. The breasts are heterogeneously dense, which may obscure small masses.

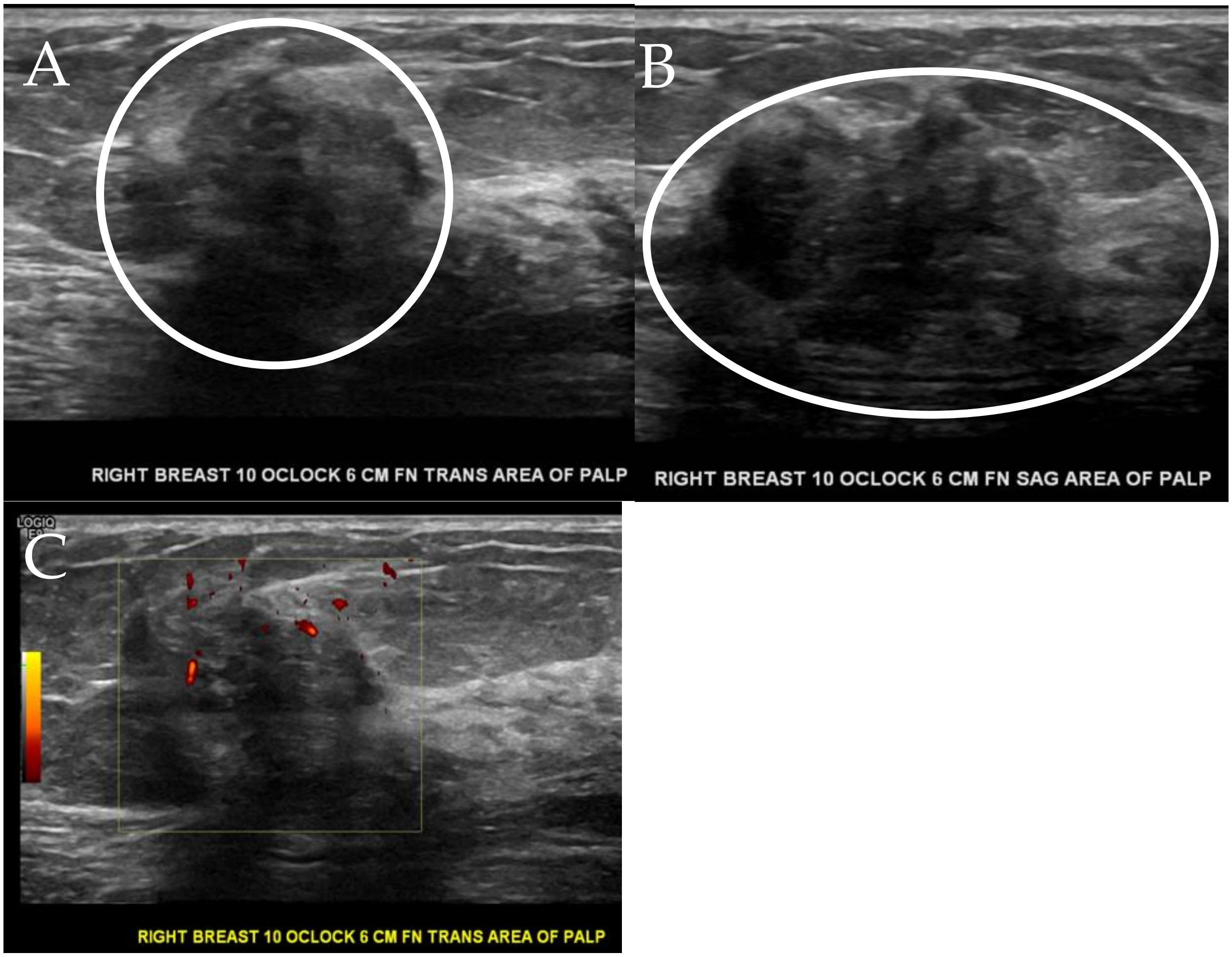

Figure S4. Right breast grayscale transverse (A) and sagittal (B) and color Doppler (C) images demonstrate an irregular, hypoechoic mass (circles) with microlobulated margins measuring 20 x17 x 25 mm seen in the right breast at 10 o'clock located 6 centimeters from the nipple. There is a combined posterior acoustic effect associated with the lesion. The mass had internal vascularity.

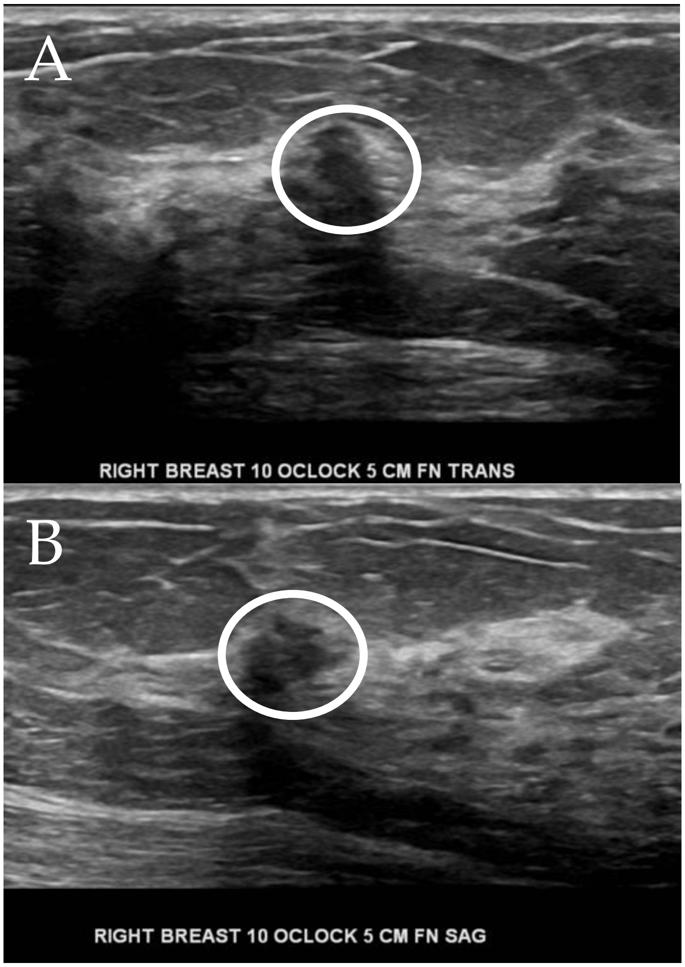

Figure S5. Right breast grayscale transverse (A) and sagittal (B) ultrasound also shows an additional similar mass (cirlces) which is also irregular, hypoechoic with microlobulated and spiculated margins measuring 5 x 5 x 8 mm seen in the right breast at 10 o'clock located 5 centimeters from the nipple. There is a combined posterior acoustic effect associated with the lesion. However, unlike the previous mass, there is no vascularity.

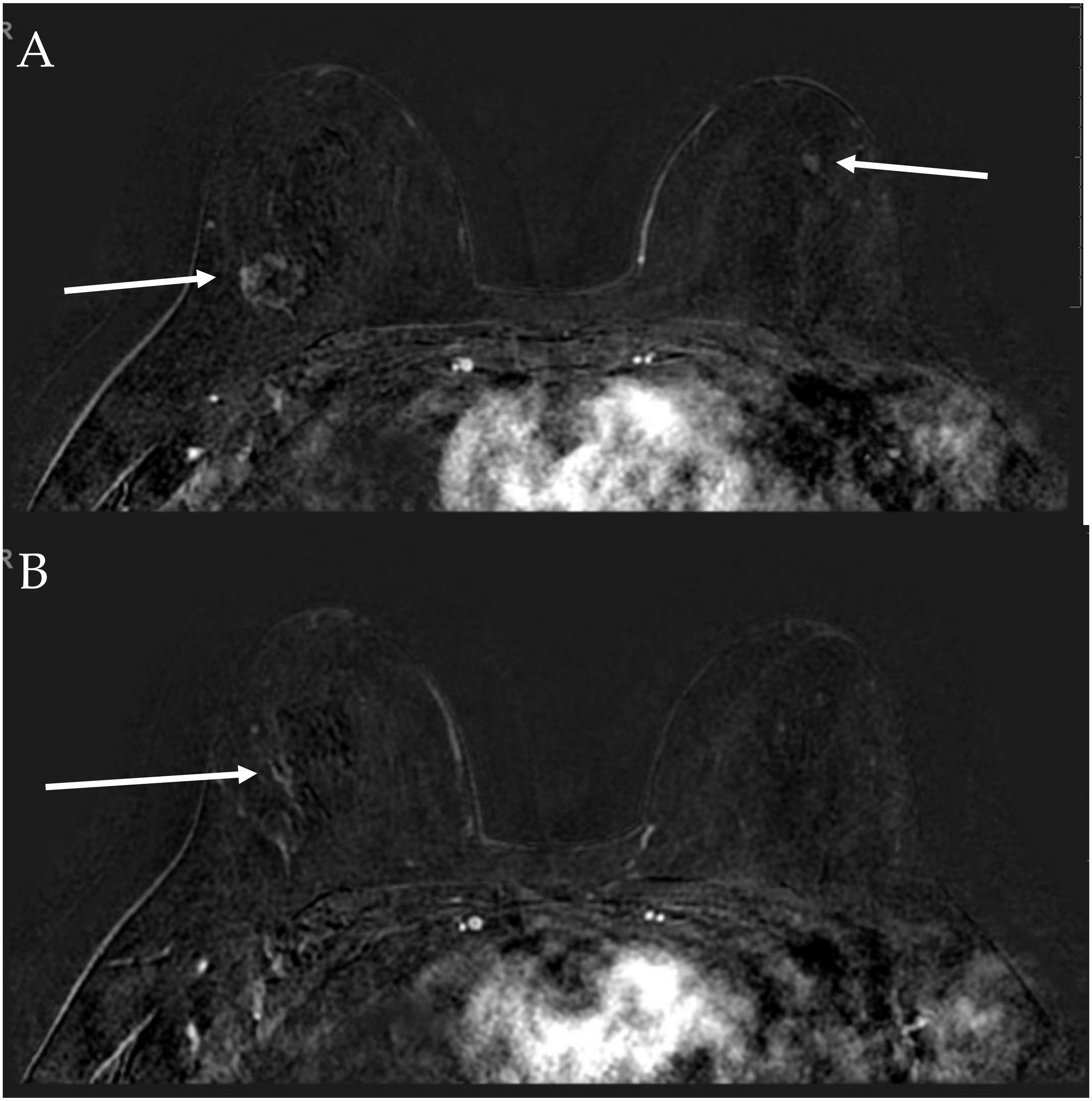

Figure S6. Two axial subtraction breast MRI images of the bilateral upper breasts. A: An irregular, enhancing 2.1 x 2.4 x 2.2 cm mass (arrow) which represents known IDC of the upper outer right breast, posterior depth. B: Incidental enhancing mass (arrow) of the left breast was subsequently biopsy proven to be benign. Bottom image demonstrates faint 1.9 cm linear non-mass enhancement extending anteriorly from the right cancer. No enlarged axillary nodes.

CASE 3: Patient is a 59-year-old female with left breast invasive ductal carcinoma (ER+,PR+, HER2-) status post lumpectomy and chemoradiation who was diagnosed with residual disease as well as mediastinal and internal mammary lymphadenopathy.

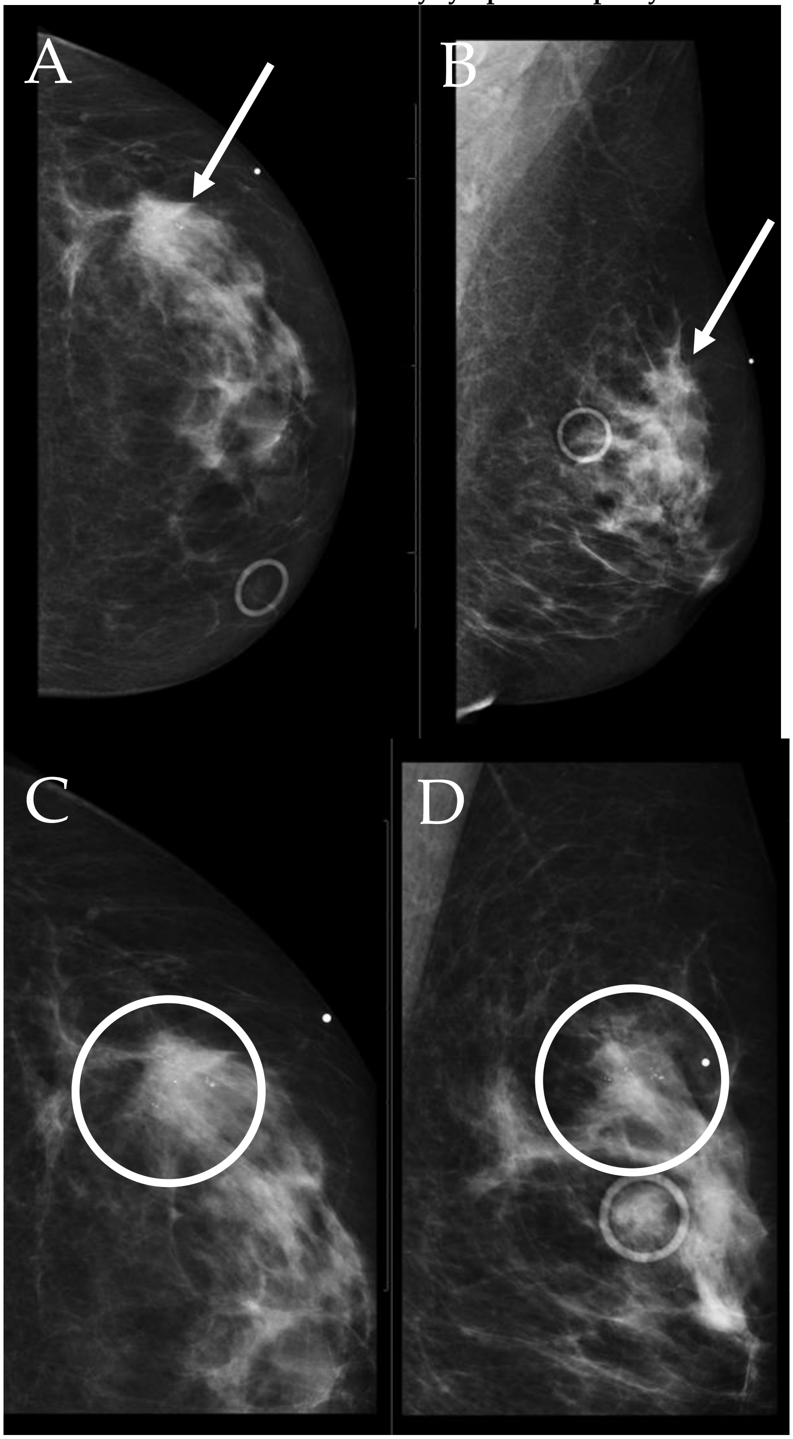

Figure S7. Left breast 2D CC (A) and MLO (B) full-field and magnification CC (C) and ML (D) views demonstrate a focal asymmetry (arrows) with associated coarse heterogeneous calcifications seen in the upper outer quadrant of the left breast (large circles) at the site of palpable concern as denoted by the radiodense skin marker. The radiodense circular marker (small circle) represents a skin mole.

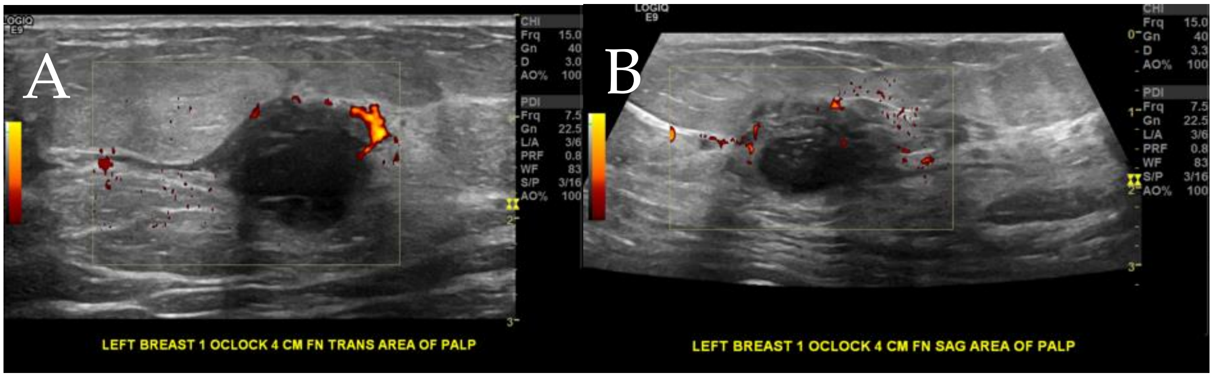

Figure S8. Left breast color Doppler transverse (A) and sagittal (B) ultrasound demonstrates an irregular not parallel hypoechoic mass with angular margins measuring 16 x 20 x 27 mm seen in the upper outer quadrant of the left breast. The echotexture of the mass is heterogeneous and there is a combined posterior acoustic effect associated with the lesion. Vessels are noted within the mass.

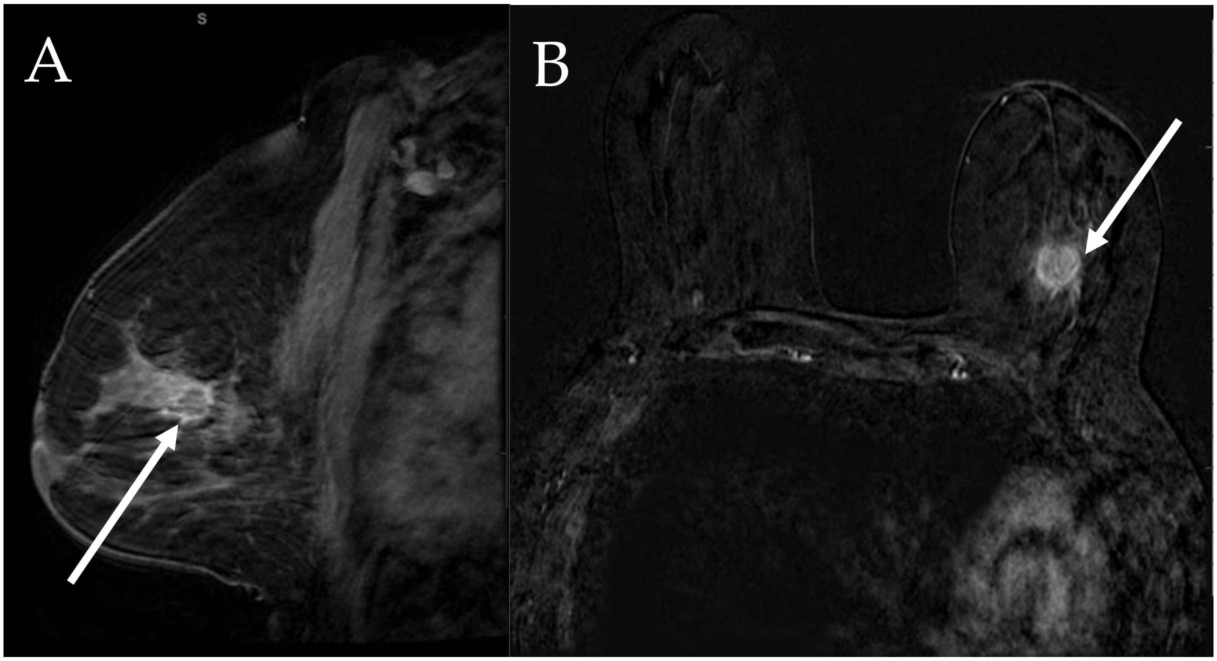

Figure S9. Left breast sagittal (A) and axial (B) T1 post-contrast subtraction images were obtained which demonstrated an 18 x 20 x 14 mm irregular mass (arrows) with spiculated margins measuring approximately 27 mm from the lateral skin and 17 mm from the pectoralis muscle. This is seen inferior and medial to the post-lumpectomy surgical distortion at the 12:00 position, posterior depth. Biopsy was recommended confirming the breast cancer diagnosis.

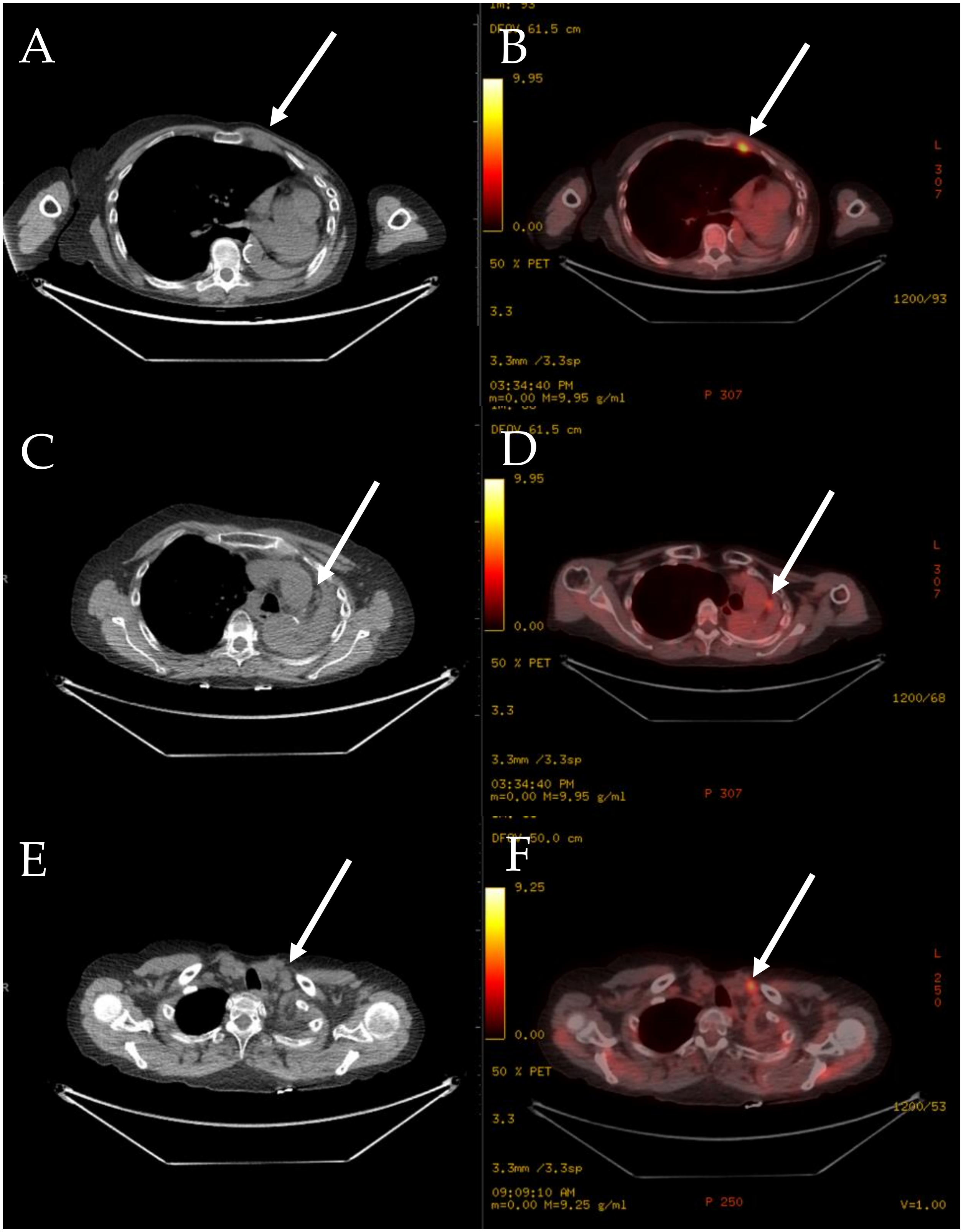

Figure S10. Axial CT (A) and corresponding PET-CT (B) at the level of the mediastinum (incidentally with chronic leftward deviation) demonstrate enlarged hypermetabolic left internal mammary node (arrow). Axial CT (C) and corresponding PET-CT (D) near aortic arch demonstrate a hypermetabolic mediastinal

node (arrows), suspicious for metastasis. Axial CT (E) and corresponding PET-CT (F) at level of clavicles shows an enlarged hypermetabolic left supraclavicular node (arrows). The supraclaivular node was later biopsy-proven to be metastatic. The internal mammary and mediastinal nodes were presumed metastatic.

CASE 4: 51-year-old female was diagnosed with right breast invasive ductal carcinoma (ER+, PR+, HER2-)

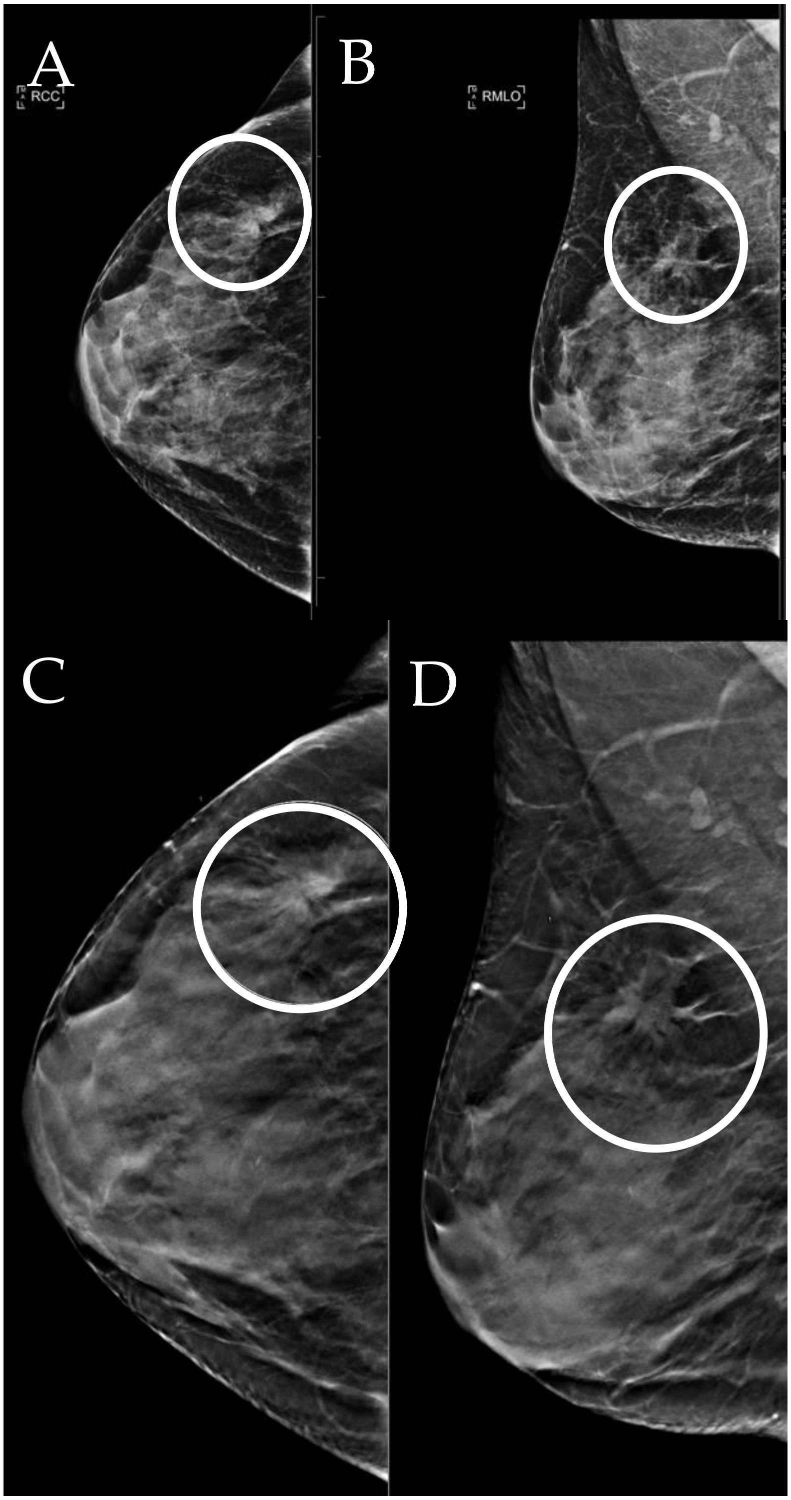

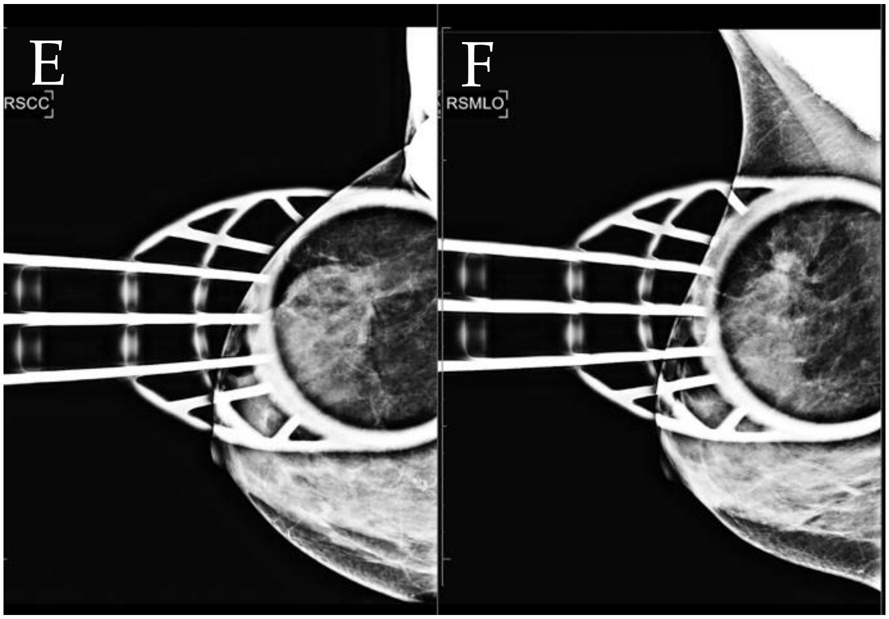

Figure S11. Right breast 2D CC (A) and MLO (B) as well as tomosynthesis images from CC (C) and MLO (D) views demonstrate architectural distortion (circles) in the upper outer quadrant. Spot CC (E) and MLO (F) views show a mass with associated architectural distortion in the upper outer quadrant of the right breast.

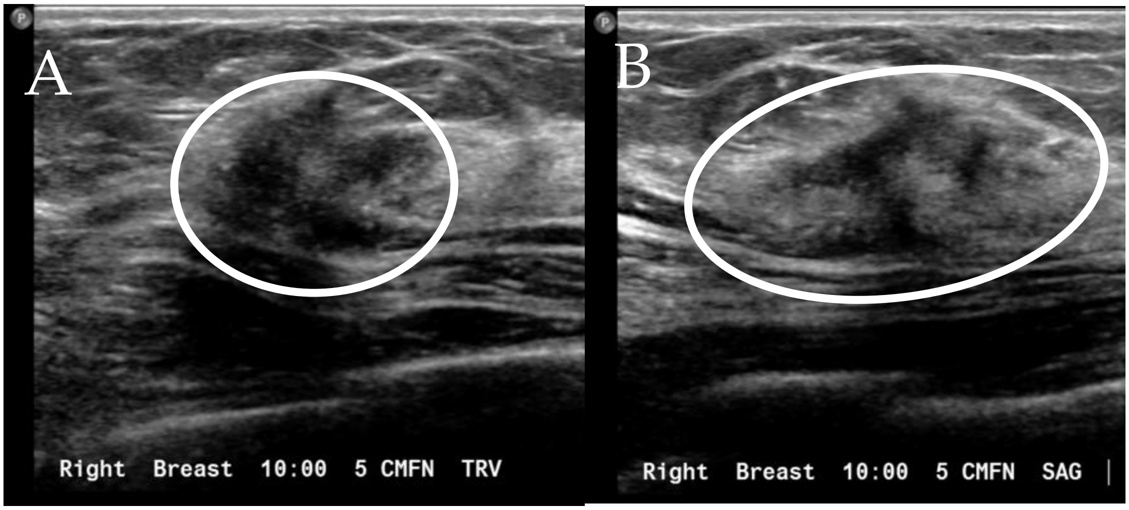

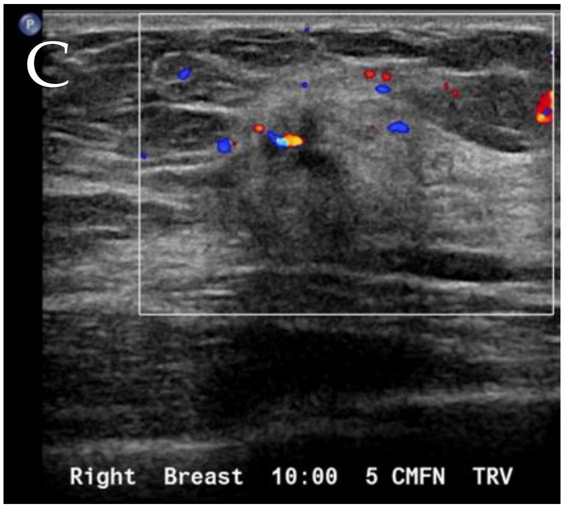

Figure S12. Right breast transverse (A) and sagittal (B) grayscale images as well as transverse color Doppler (C) images demonstrate a heterogeneously hypoechoic, irregular mass (circles) with internal vascularity which measures a 1.9 x 1.8 cm at 10:00, 5 cm from nipple. This was the biopsy proven invasive ductal carcinoma.

CASE 5: 55-year-old female with BRCA 1 mutation has a history of right invasive ductal carcinoma (ER+, PR+, HER-2+) and axillary metastasis status post lumpectomy and axillary lymph node dissection with subsequent chemoradiation. Recurrence of invasive ductal carcinoma in the right breast with the same molecular characteristics led to bilateral mastectomies. Eventually metastasis to her lymph nodes within her neck and chest were also found.

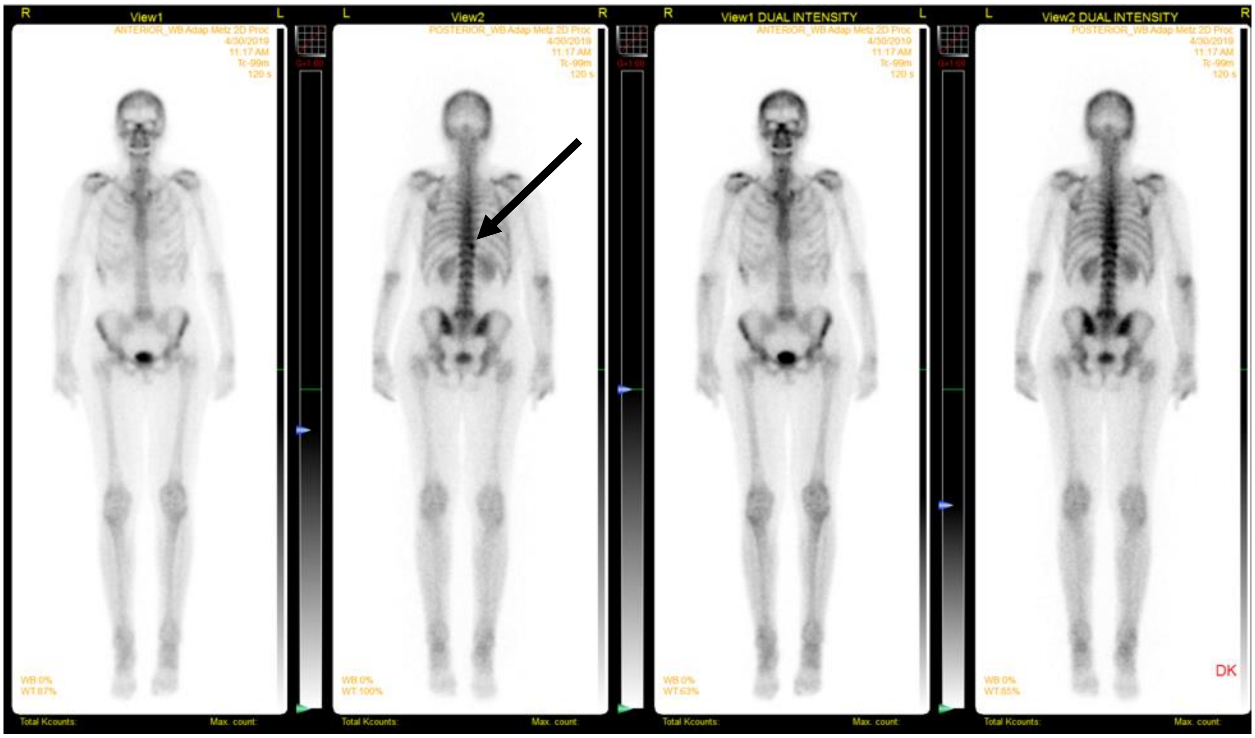

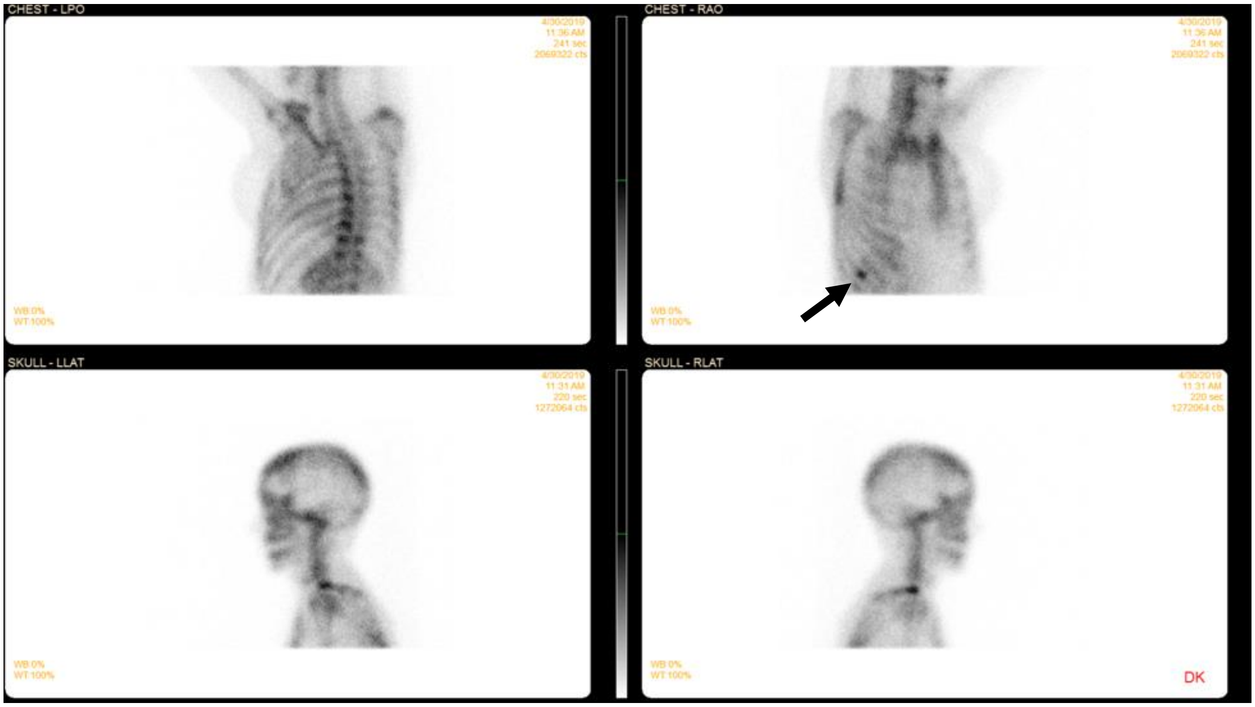

Figure S13. Bone Scan with Tc-99m MDP: Delayed skeletal phase planar whole body and spot images of the chest and skull were obtained. Mild radiotracer uptake noted in an anterior right ninth rib (short arrow) and T11 vertebral body, consistent with osseous metastases (long arrow).

CASE 6: 41-year-old female with history of left breast invasive mammary carcinoma (HER2 positive, ER weakly positive, PR negative) with metastasis to lung, liver, and bone.

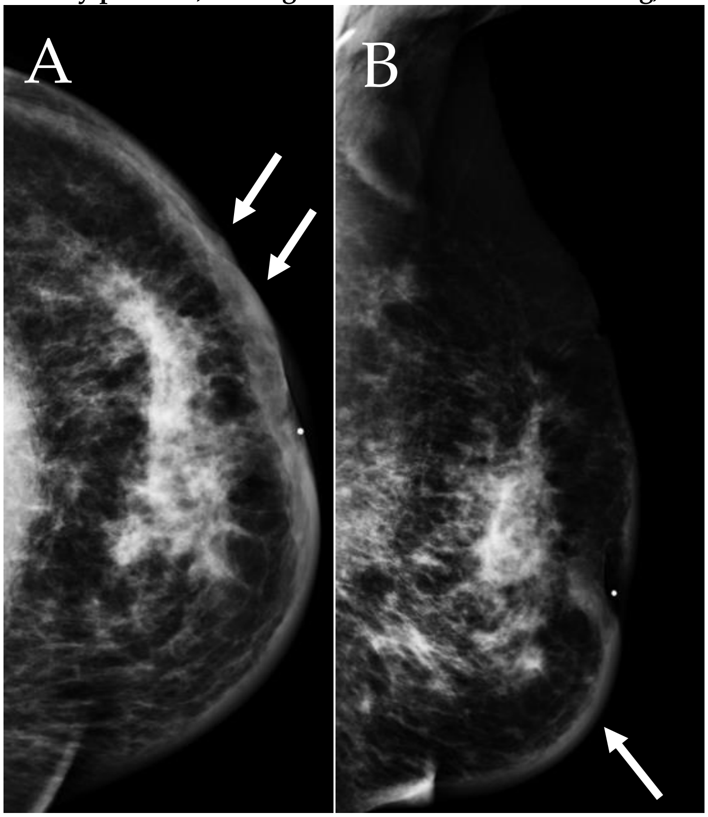

Figure S14. Left breast 2-D CC (A) and MLO (B) views demonstrate a global asymmetry with trabecular and skin thickening (arrows) and associated architectural distortion. Overall decreased breast size noted.

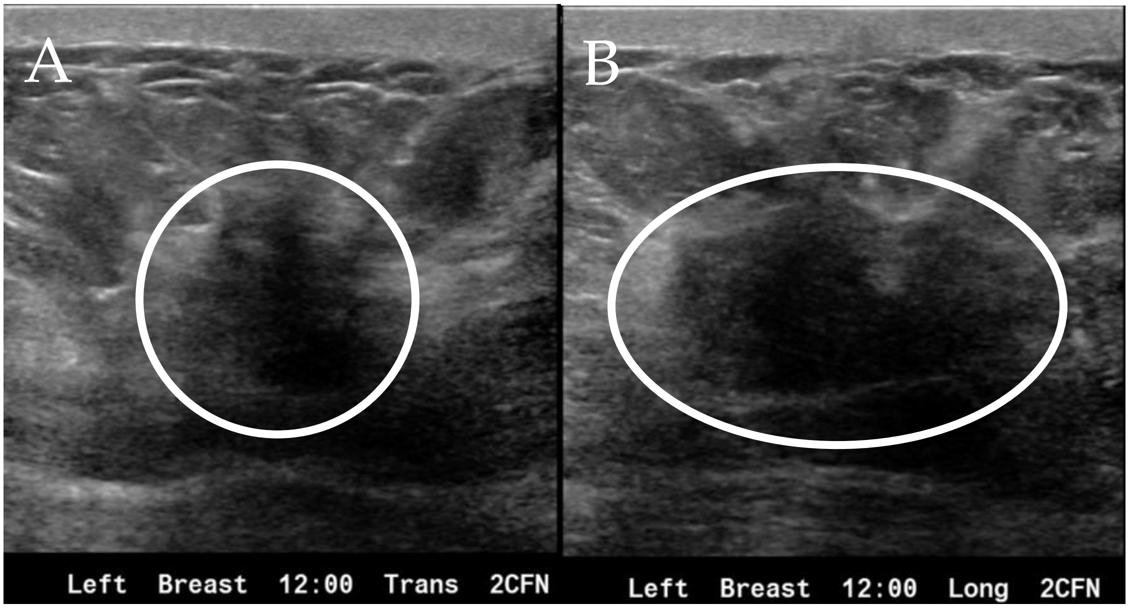

Figure S15. Grayscale ultrasound of the left breast in the transverse (A) and sagittal (B) dimensions demonstrate a hypoechoic irregular not parallel mass (circles) measuring up to 3.2 cm at 12:00, 2 cm from nipple.

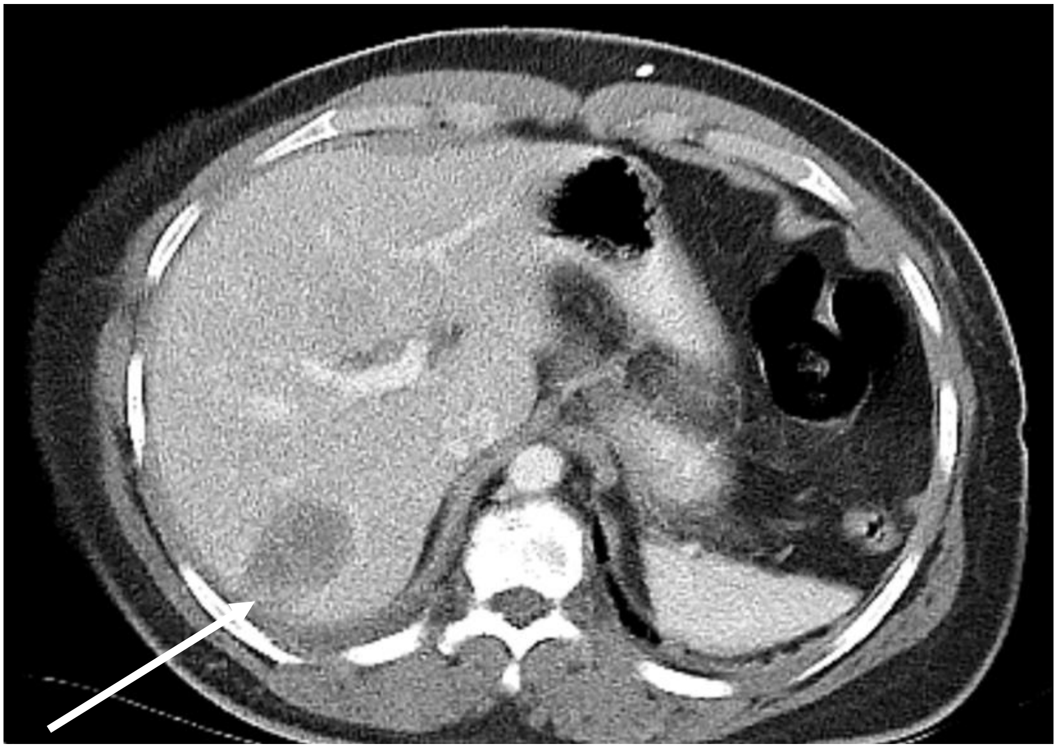

Figure S16. Axial contrast-enhanced CT slice of the liver showed multiple hypoattenuating liver lesions suggestive of metastatic disease. Largest lesion located in the right hepatic lobe measures 4.1 x 2.4 cm (arrow).

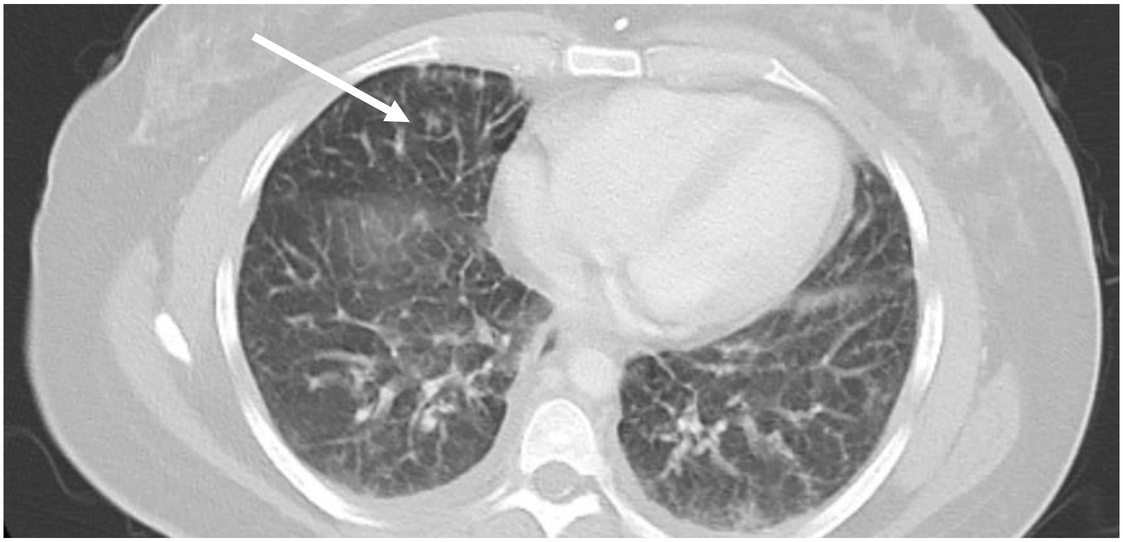

Figure S17. CT chest with lung window demonstrated multiple, bilateral, pulmonary nodules measuring between 5 to 7 mm, suggestive of pulmonary metastasis

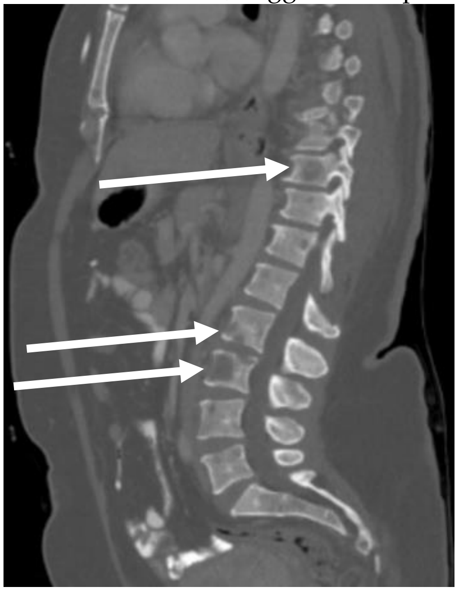

Figure S18. Bone window of CT abdomen and pelvis at midline demonstrated numerous lytic lesions throughout the entire spine , suggestive of metastatic disease with arrows pointing to the larger lesions.

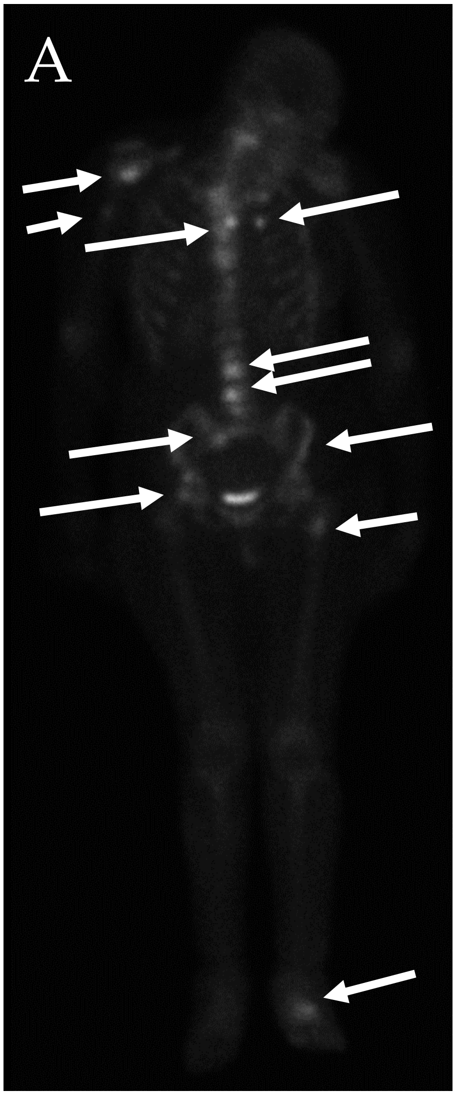

Figure S19. Bone scan using Technetium 99m methylene diphosphonate (Tc-99m MDP) full body scan demonstrated multifocal osseous metastases with abnormal activity noted throughout the axial skeleton involving multiple thoracic and lumbar spine vertebral levels, bilateral pelvis, right femoral head, proximal left femoral metadiaphysis, right shoulder, right proximal humeral diaphysis and multiple posterior left ribs. Focal moderate uptake in the lower cervical spine and left ankle was also suspicious. Arrows point to several of the aforementioned areas.

CASE 7: 78-year-old female with BRCA 1 mutation diagnosed with invasive ductal carcinoma of the left breast (ER-, PR-, HER2neu-).

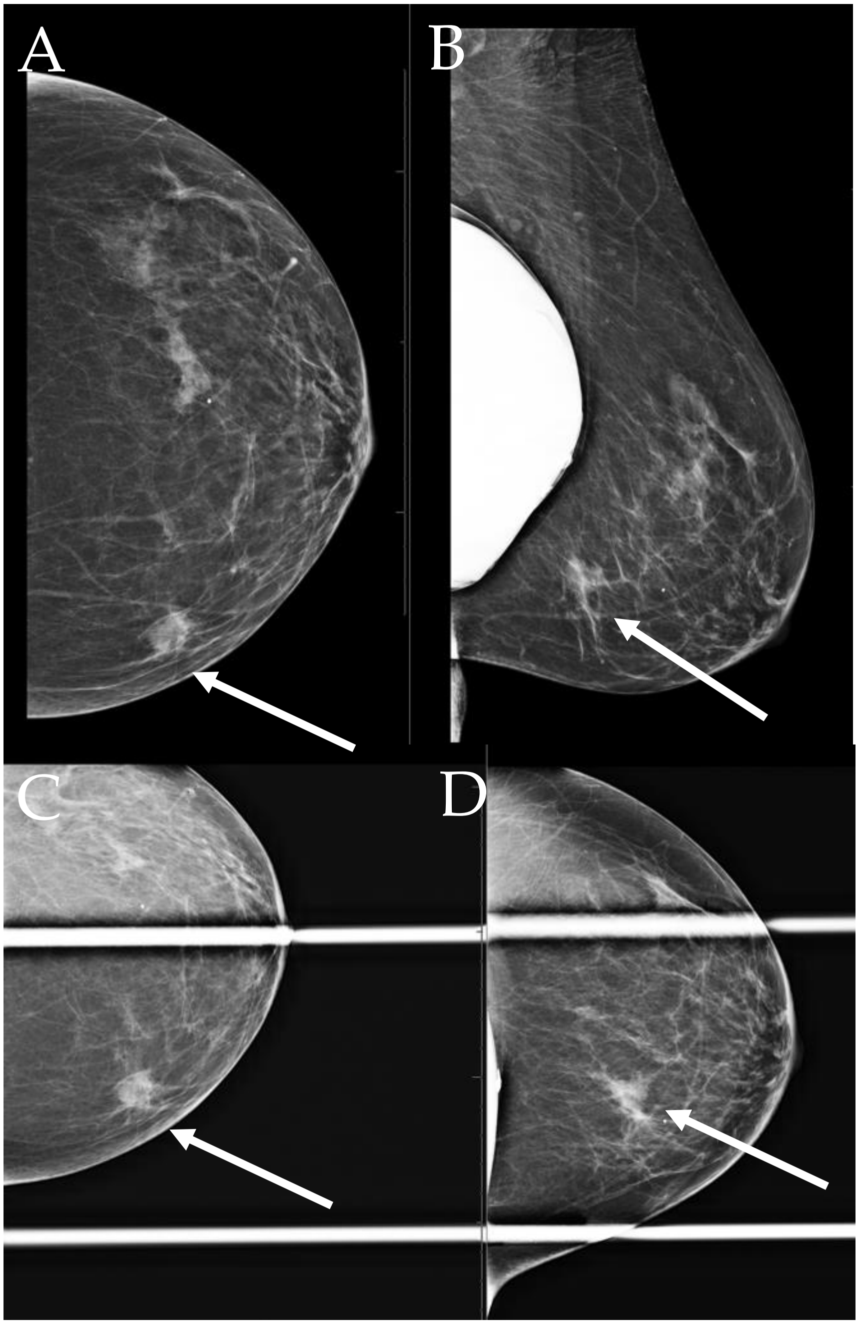

Figure S20. Left breast 2D CCID (A) and MLO (B) views demonstrate a retropectoral silicone gel implant and an irregular mass (arrows) seen in the posterior depth lower inner quadrant. Dedicated CC (C) and MLO (D) spot views showed a high density, irregular mass with microlobulated margins . Associated architectural distortion in the posterior depth upper inner quadrant of the left breast is not well seen on these 2D images.

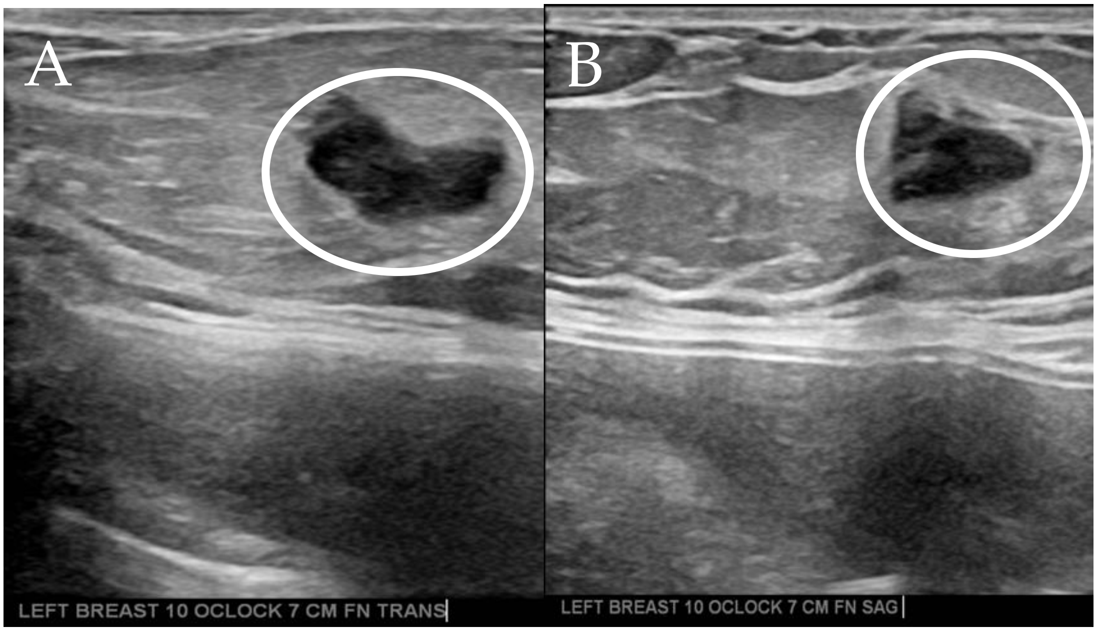

Figure S21. Left breast Grayscale US with transverse (A) and sagittal (B) views demonstrated a hypoechoic, irregular not parallel mass with indistinct margins measuring 15 x 10 x 15 mm in the left breast at 10:00 7cm from the nipple (circles).

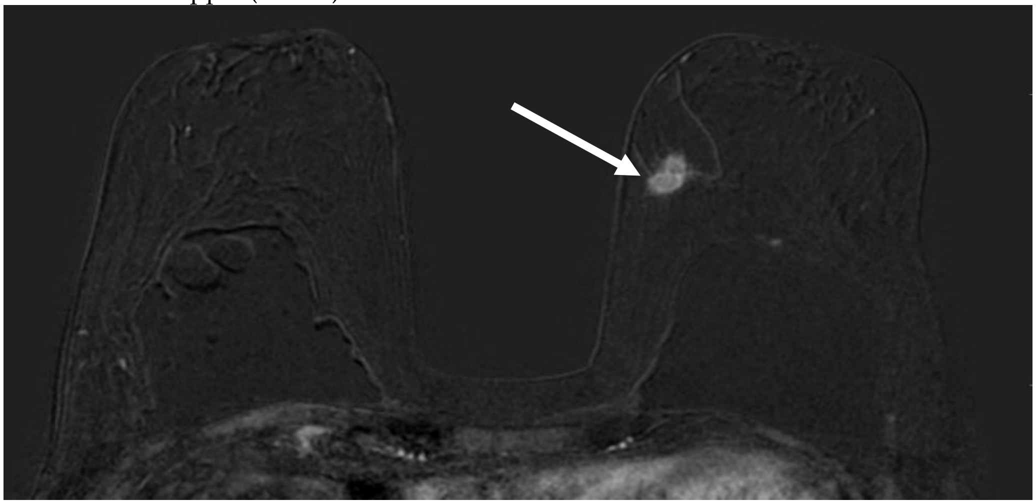

Figure S22. Axial subtraction breast MRI demonstrates an enhancing irregular mass with spiculated margins in the left breast upper inner quadrant (arrow), consistent with known invasive ductal carcinoma grade 3, measures 14 x 16 x 16 mm, demonstrating washout kinetics. Mass is within 5 mm of the medial skin.

CASE 8: 39 y/o female who presented with a palpable finding was diagnosed with right triple negative IDC.

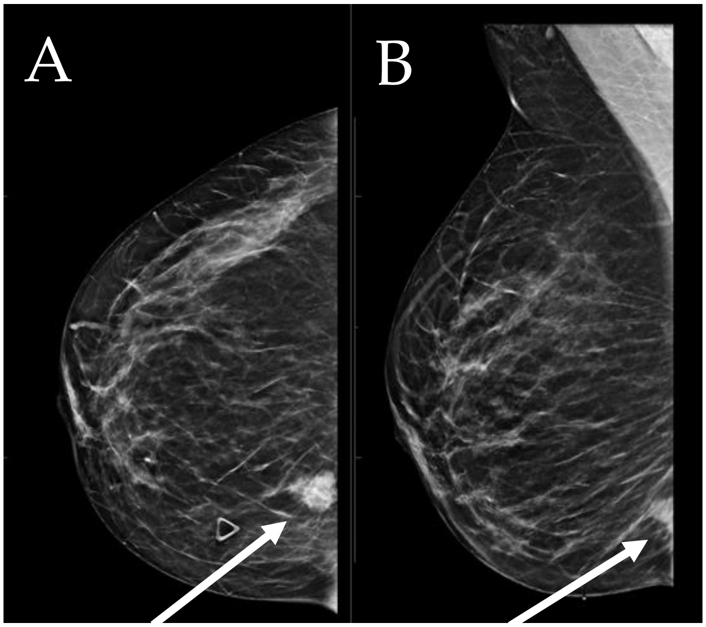

Figure S23. Right breast 2D CC (A) and MLO (B) views demonstrate an irregular mass (arrows) in the lower inner quadrant posterior depth which corresponds to a site of palpable concern marked by triangular marker.

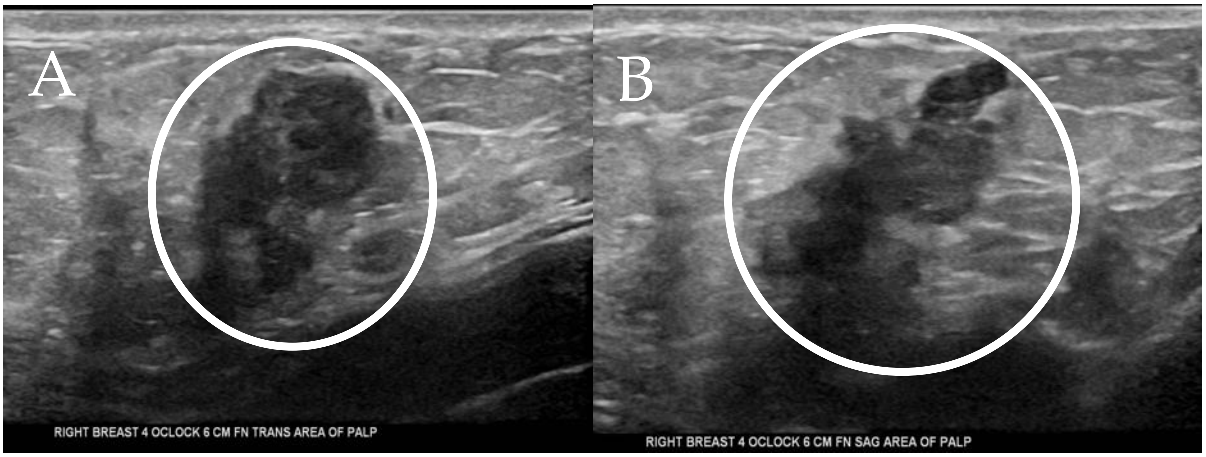

Figure S24. Right breast greyscale transverse (A) and sagittal (B) US shows an irregular, hypoechoic mass in the right breast 4:00 6 cm from nipple (circles) which was the biopsy-proven ER/PR positive HER-2 negative high-grade invasive ductal carcinoma with high-grade DCIS.

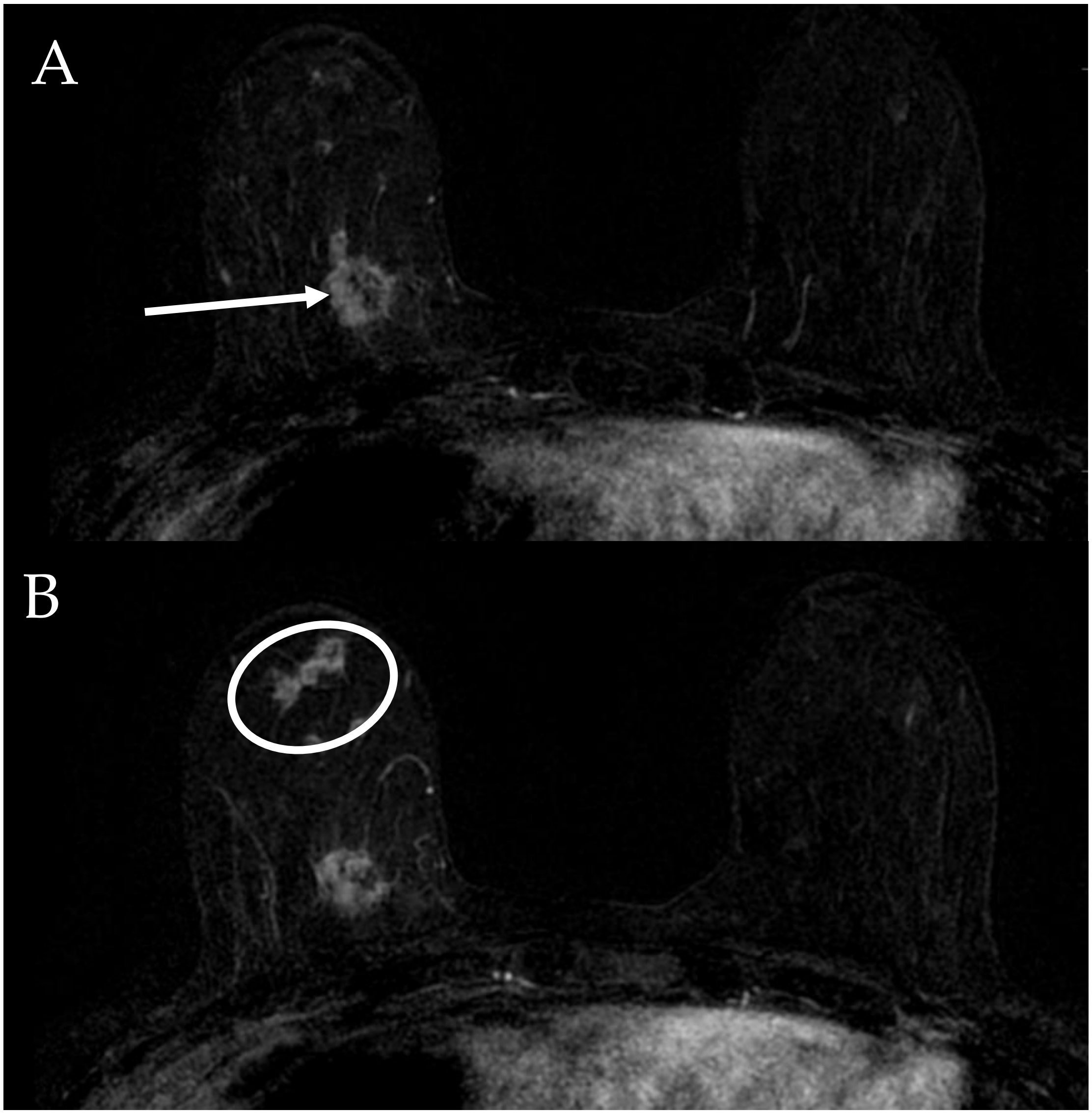
A
B

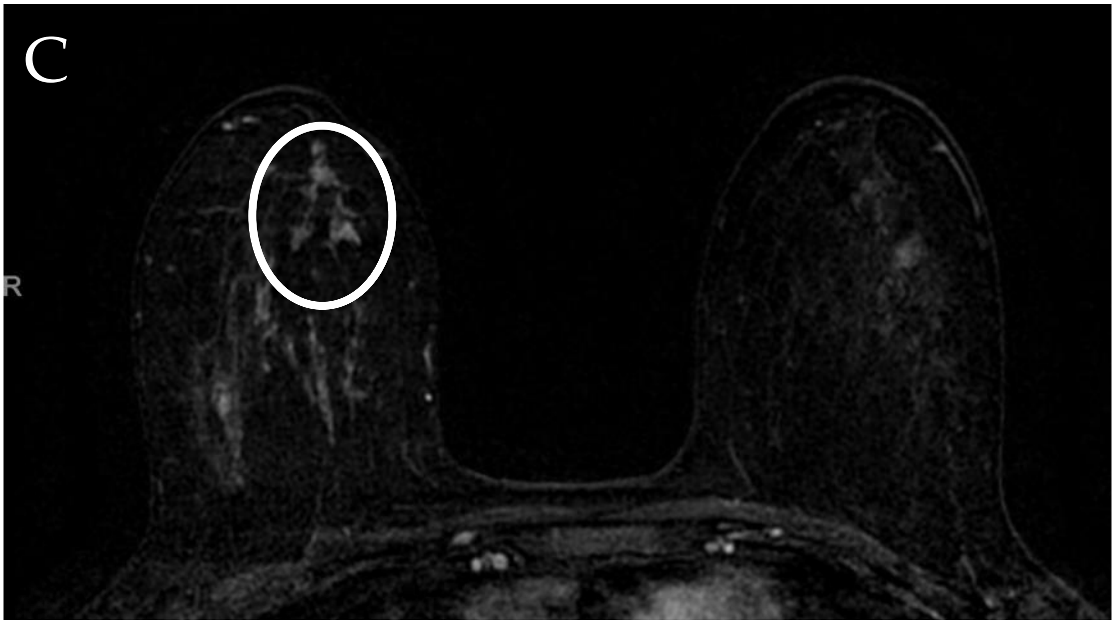

Figure S25. A: Axial subtraction MRI shows a small irregular spiculated mass which measures 2.3 cm in its greatest diameter in the lower inner quadrant of the right breast. Signal void within the mass is consistent with the biopsy clip. This is the known malignancy. B: Axial subtraction MRI shows a second irregular enhancing mass with associated architectural distortion which measures approximately 2 cm in its greatest diameter in the 6:00 position anterior to the aforementioned mass, separated by approximately 4 cm. This was biopsied under MR guidance and was found to represent IDC and DCIS. Given multiple areas of abnormal enhancement, this is consistent with multicentric malignancy and mastectomy was performed. C: Axial subtraction MRI shows non-mass enhancement in the upper inner quadrant at anterior depth of the right breast, which is asymmetric from the contralateral breast. If breast conservation was being contemplated, MRI-guided biopsy was recommended, but patient eventually underwent mastectomy.

CASE 9: 60-year-old female with invasive ductal carcinoma ER- PR- HER2+ s/p chemoradiation who presented with a new palpable finding in the right breast and was found to have biopsy proven recurrence and metastatic disease including mediastinal lymphadenopathy and peritoneal carcinomatosis.

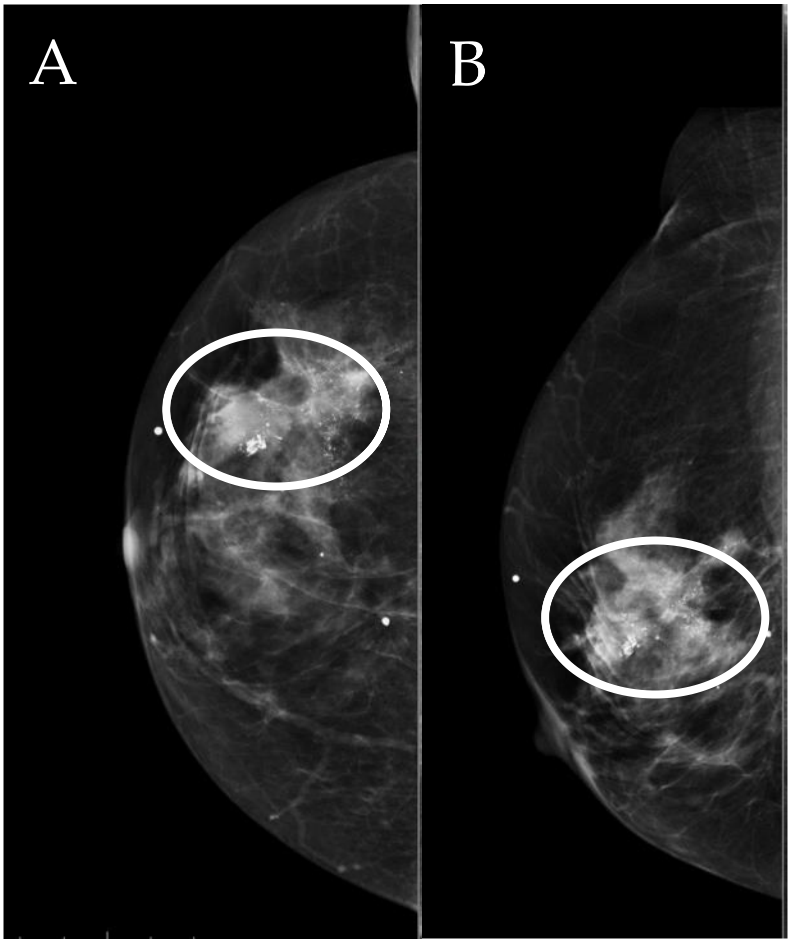

Figure S26. Right breast CC (A) and MLO (B), irregular mass with associated fine pleomorphic calcifications (circles) seen in the upper outer quadrant of the right breast at middle depth .

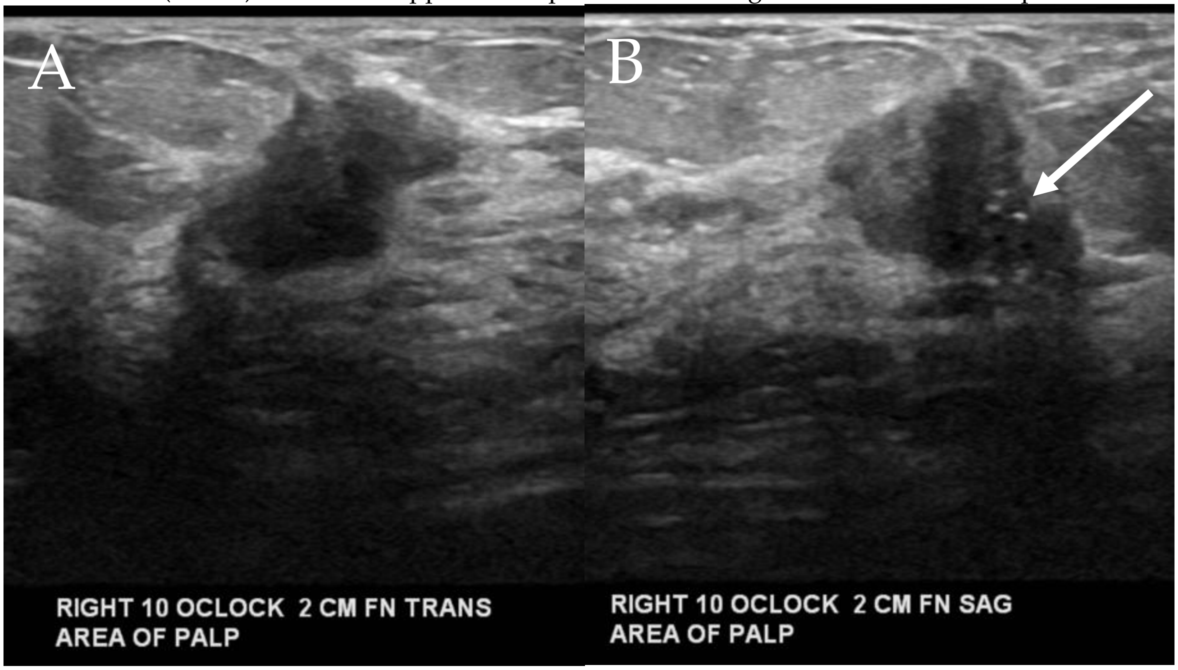

Figure S27. Grayscale targeted ultrasound of the right breast in the transverse (A) and sagittal (B) dimension demonstrates one mass in the right breast at 10 o'clock. This was one of several grouped masses seen in the right breast at 9 o'clock and 10 o'clock with aggregate of the masses measuring 4.4 x 1.3 cm. Internal specular reflectors suggestive of calcifications (arrow).

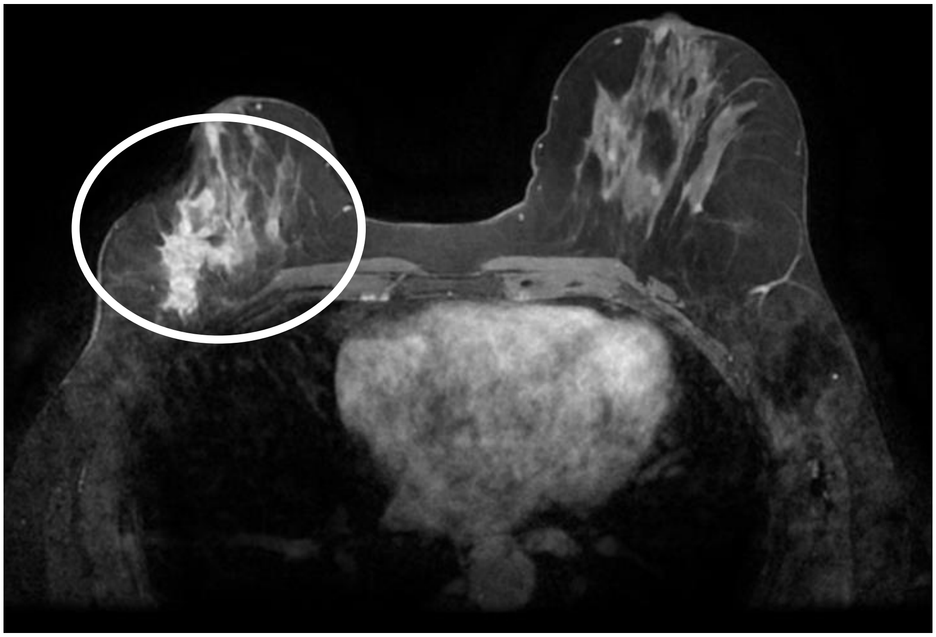

Figure S28. Axial T1 dynamic contrast enhanced (DCE) fat subtracted (FS) image at the level of the nipple demonstrates an irregular large mass and associated nonmass enhancement in the right central and lateral breast extending nearly from chest wall to nipple measuring at least 24 x 76 x 39 mm, involving the nipple and approximately 5 mm from the underlying chest wall.

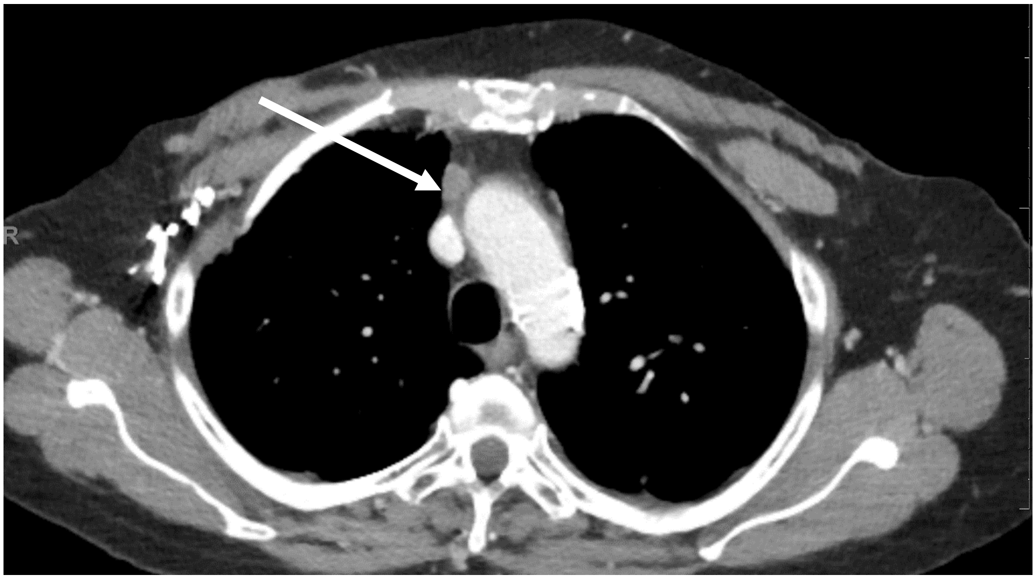

Figure S29. Axial contrast-enhanced CT at the level of the aortic arch demonstrates enlarged anterior mediastinal lymph node, which was biopsy proven metastasis. Presumed metastatic pulmonary nodules, which were too small to be biopsied, are not well visualized in this window.

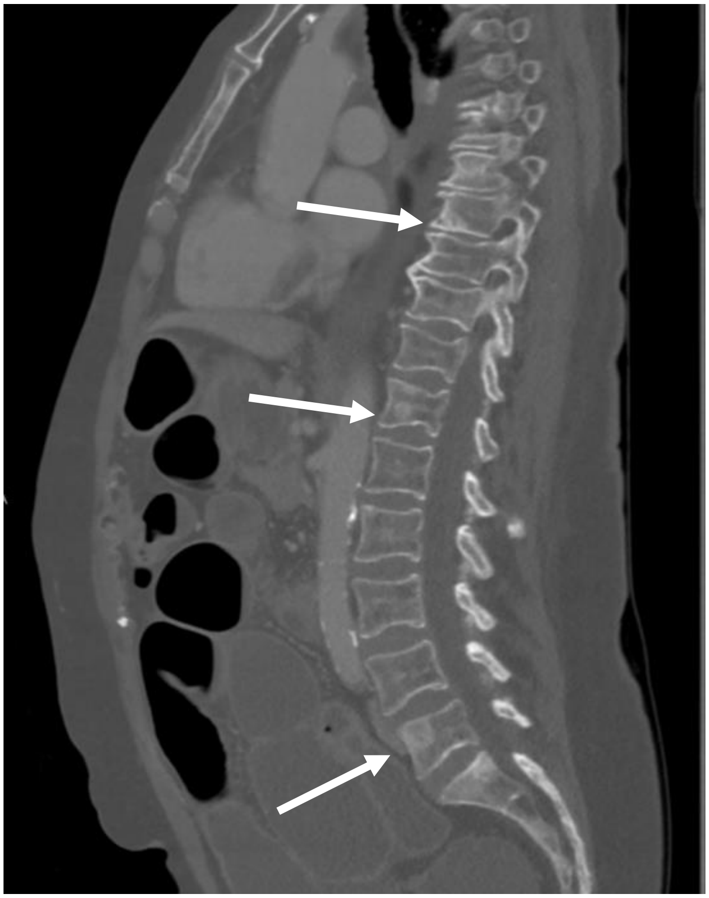

Figure S30. Sagittal CT of the abdomen and pelvis (bone window) at the midline demonstrates multiple sclerotic lesions within the vertebral bodies including T8, T10, T12, L1, and L5 (arrows point towards T8, T12, and L5 lesions), presumed osseous metastases

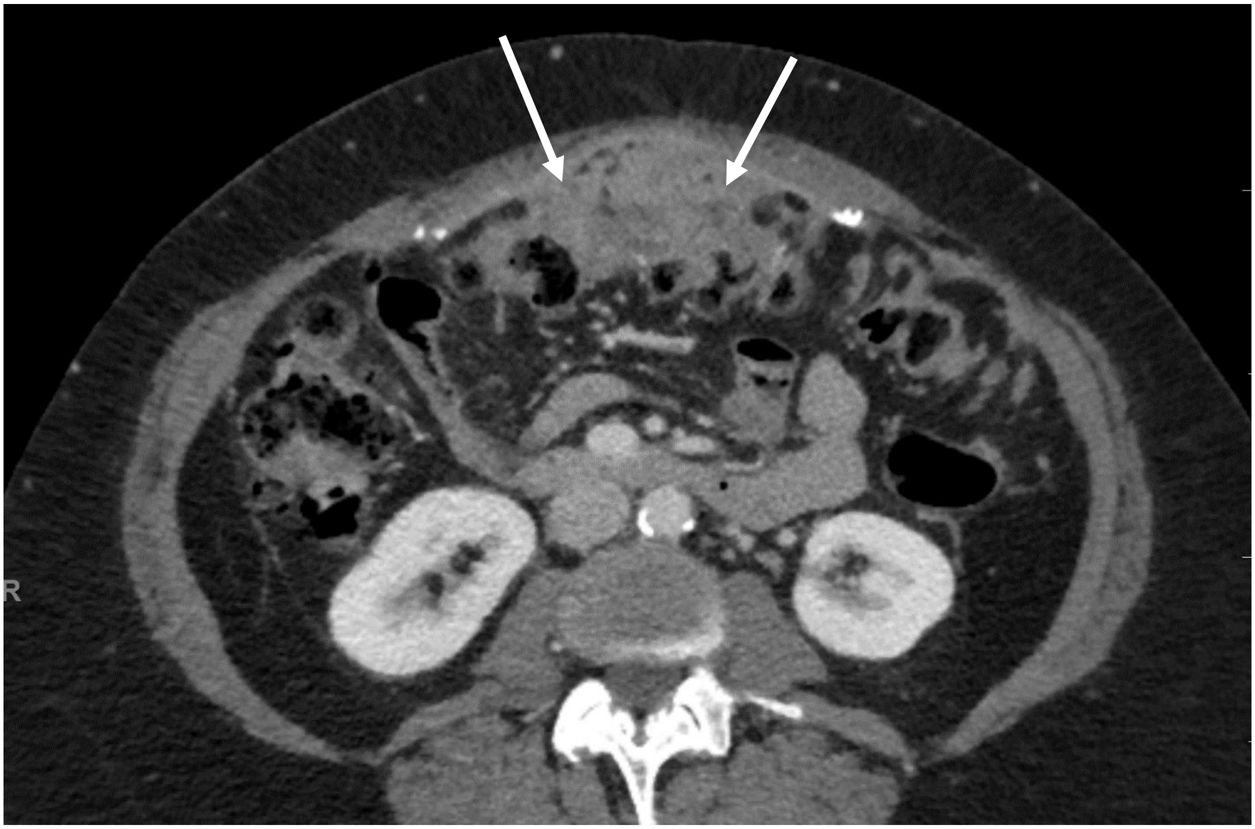

Figure S31. Contrast-enhanced CT of the abdomen at the level of the lower pole of the kidneys demonstrates large volume omental caking (arrows) along the anterior abdominal well and abutting the transverse colon suggestive of carcinomatosis and later biopsy proven

CASE 10: 57-year-old female who was diagnosed with right invasive ductal carcinoma ER-/PR-/HER2+

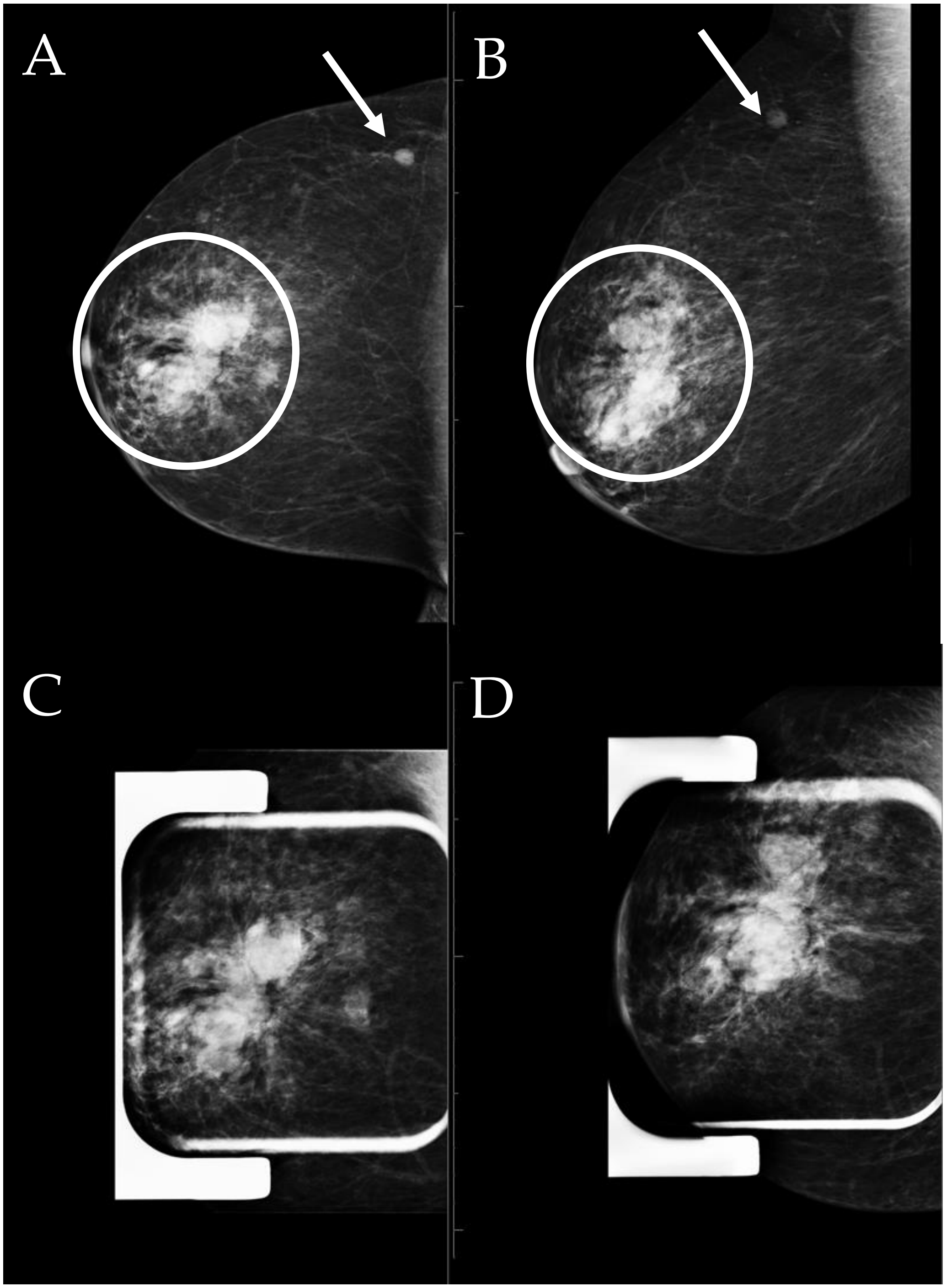

Figure S32. Right breast 2D CC (A) and MLO (B) views as well as spot compression CC (C) and MLO (D) views demonstrate a high density, irregular mass measuring 131 x 95 x 85 mm with indistinct margins seen in the right breast in the central, upper outer and upper inner anterior depth (circles). The mass correlates with the palpable abnormality in the right breast. There is also an oval mass seen in the posterior depth upper outer quadrant of the right breast (arrows).

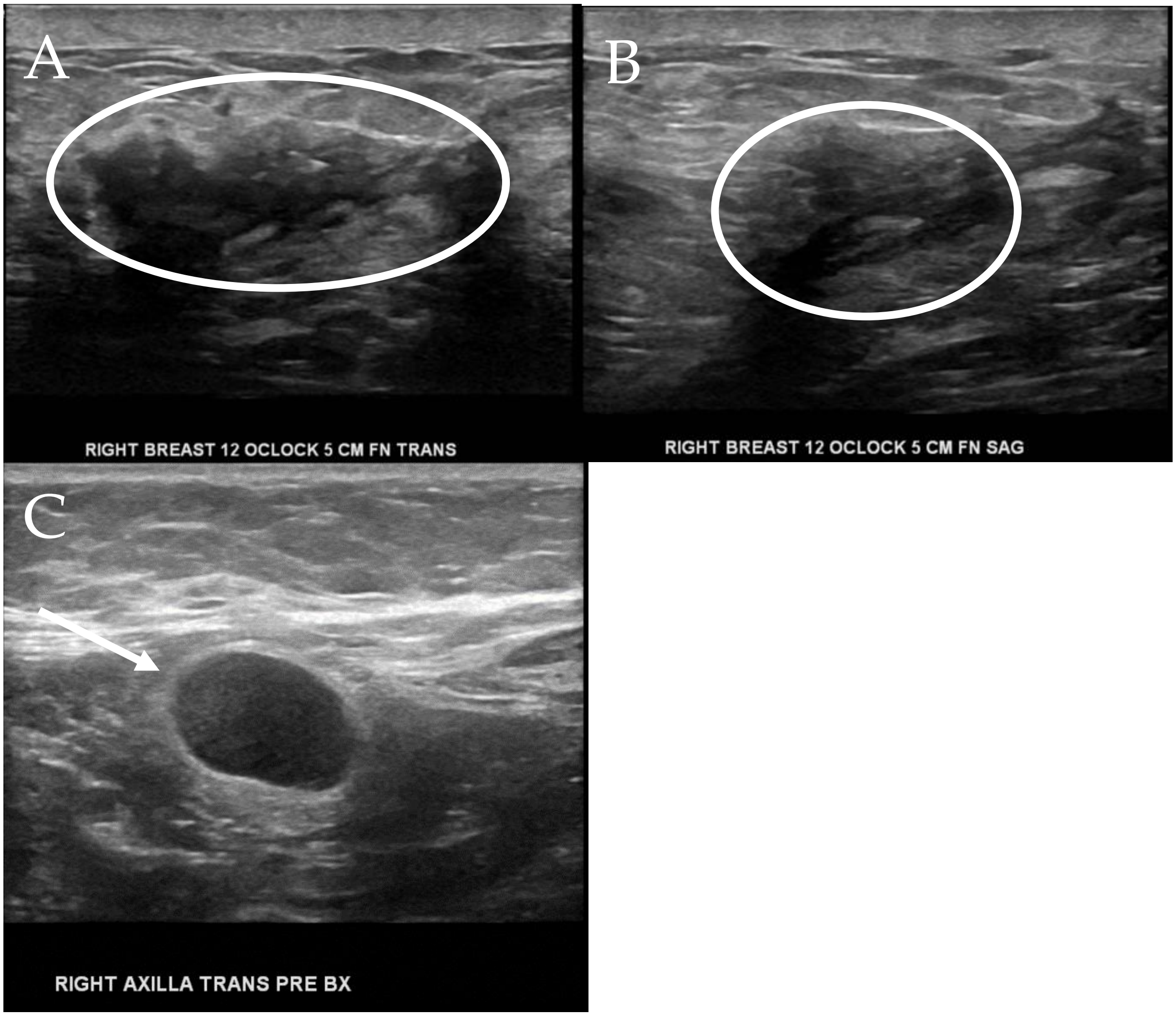

Figure S33. Right breast, greyscale transverse (A) and sagittal (B) images demonstrate a hypoechoic, irregular, parallel mass with indistinct margins measuring 34 x 11 x 18 mm seen in the right breast at 12 o'clock located 5 centimeters from the nipple. Internal echotexture is heterogeneous. This was biopsy proven invasive ductal carcinoma. There is associated skin thickening measuring at least 4 mm. Enlarged abnormal right axillary lymph node (C) with an effaced fatty hilum (arrow) was a biopsy proven metastasis.

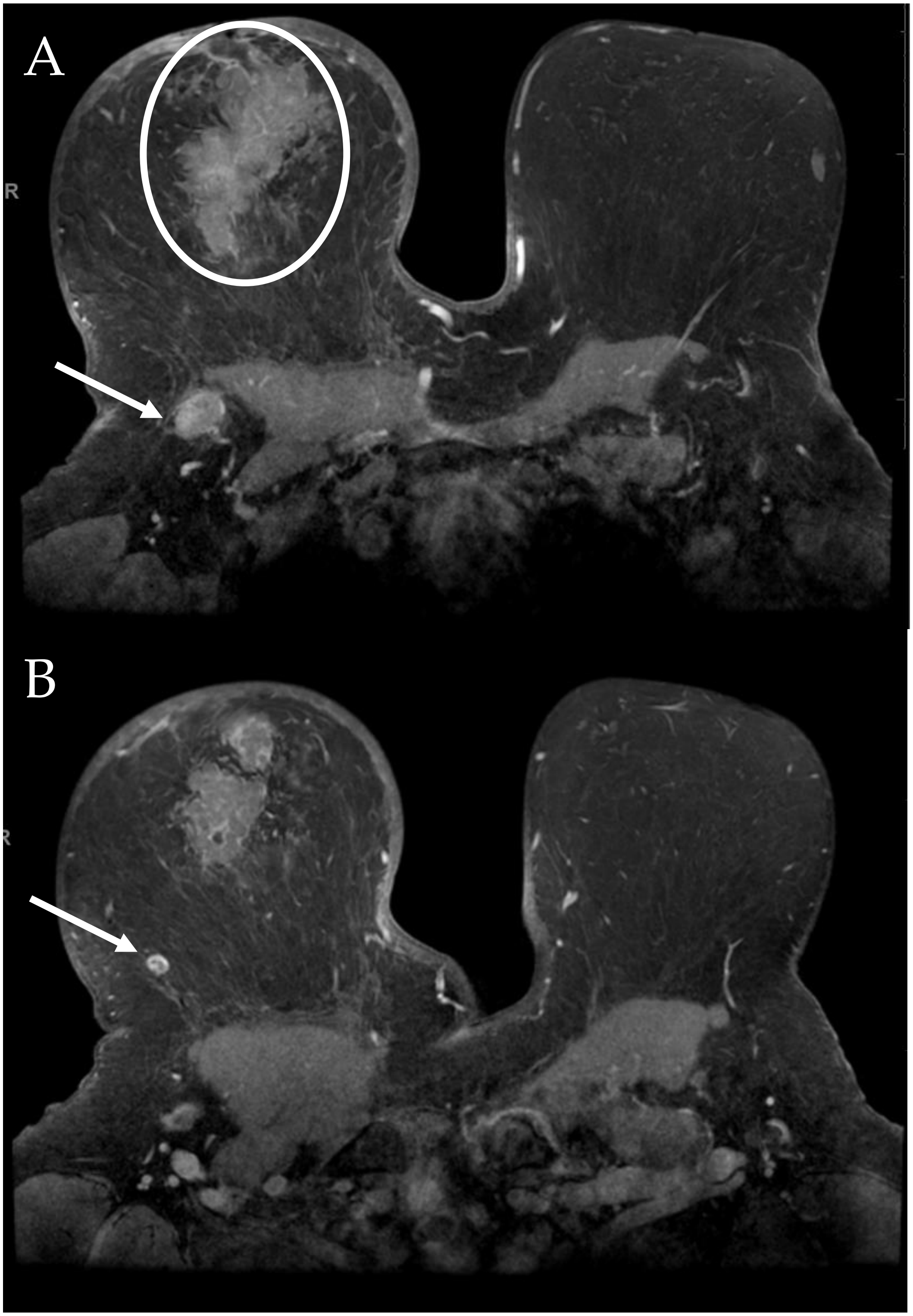

Figure S34. Right breast axial T1 FS DCE images demonstrates large irregular mass measuring at least 102 x 73 x 77 mm invading the overlying skin which represents the patient's biopsy-proven invasive ductal carcinoma (A). The mass involves the central, upper inner, upper outer, lower inner, and lower outer breast. Enlarged abnormal right axillary lymph node (arrow), later biopsy proven metastatic. Second image (B) demonstrates a satellite mass in the posterior lateral right breast (arrow).

CASE 11: 60-year-old female who presented with left breast tenderness and was diagnosed with right sided invasive ductal carcinoma and DCIS (ER-/PR-/Her2+).

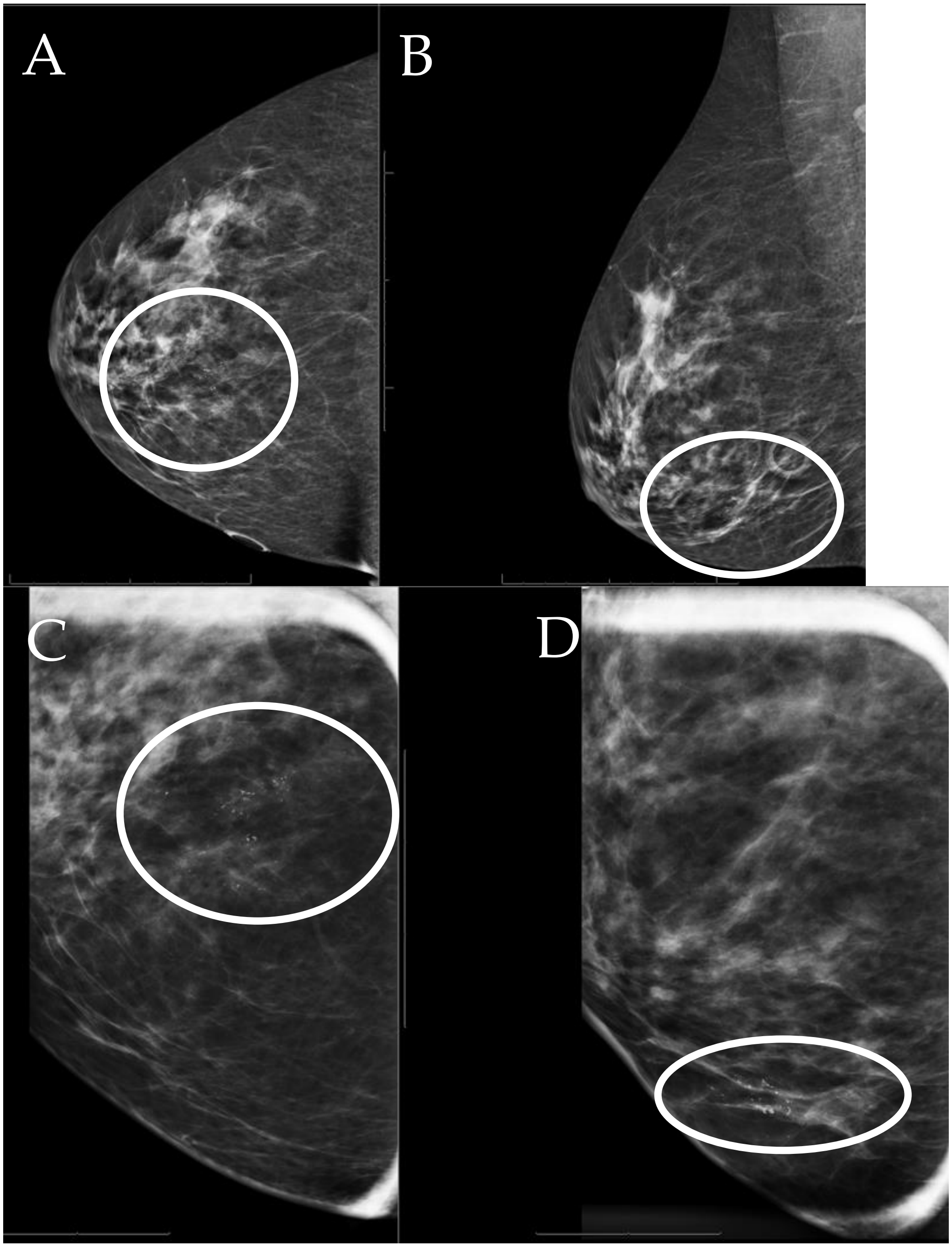

Figure S35. Right breast 2D full-field CC (A) and MLO (B) as well as magnification views of the lower inner quadrant of the right breast in the CC (C) and ML (D) views demonstrated pleomorphic calcifications (circles and ovals) in a segmental distribution at 6 o'clock middle depth.

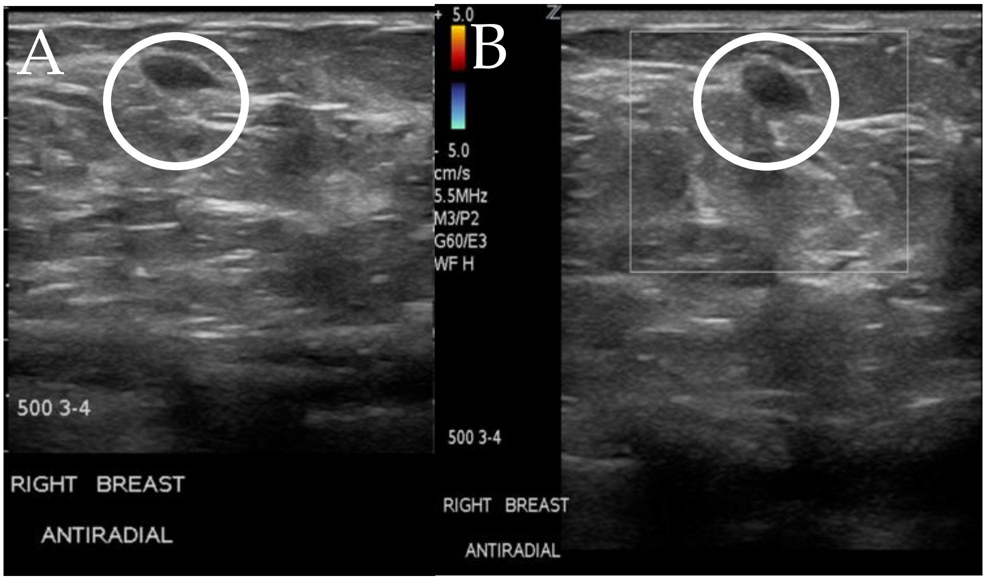

Figure S36. Right breast greyscale (A) and color Doppler (B) images were obtained only in the anti-radial direction at 5:00 which showed an 8mm hypoechoic, not circumscribed mass without internal vascularity. A core biopsy was attempted; however, it was unsuccessful, and therefore she went to the OR for excisional biopsy. This demonstrated IDC and DCIS with positive margins. She later underwent a repeat lumpectomy.
